# GPT-4 for automated sequence-level determination of MRI protocols based on radiology request forms from clinical routine

**DOI:** 10.1007/s00330-025-11888-4

**Published:** 2025-08-08

**Authors:** Robert Terzis, Kenan Kaya, Thomas Schömig, Jan Paul Janssen, Andra-Iza Iuga, Jonathan Kottlors, Simon Lennartz, Carsten Gietzen, Cansin Gözdas, Lukas Müller, Robert Hahnfeldt, David Maintz, Thomas Dratsch, Lenhard Pennig

**Affiliations:** 1 Institute for Diagnostic and Interventional Radiology, Faculty of Medicine and University Hospital Cologne, University of Cologne, Cologne, Germany; 2 Department of Diagnostic and Interventional Radiology, University Medical Center of the Johannes Gutenberg-University, Mainz, Germany

**Keywords:** Artificial intelligence, Large language models, Cardiac imaging, Neuroradiology, Magnetic resonance imaging

## Abstract

**Objectives:**

This study evaluated GPT-4’s accuracy in MRI sequence selection based on radiology request forms (RRFs), comparing its performance to radiology residents.

**Materials and methods:**

This retrospective study included 100 RRFs across four subspecialties (cardiac imaging, neuroradiology, musculoskeletal, and oncology). GPT-4 and two radiology residents (R1: 2 years, R2: 5 years MRI experience) selected sequences based on each patient’s medical history and clinical questions. Considering imaging society guidelines, five board-certified specialized radiologists assessed protocols based on completeness, quality, and utility in consensus, using 5-point Likert scales. Clinical applicability was rated binarily by the institution’s lead radiographer.

**Results:**

GPT-4 achieved median scores of 3 (1–5) for completeness, 4 (1–5) for quality, and 4 (1–5) for utility, comparable to R1 (3 (1–5), 4 (1–5), 4 (1–5); each *p* > 0.05) but inferior to R2 (4 (1–5), 5 (1-5); *p* < 0.01, respectively, and 5 (1–5); *p* < 0.001). Subspecialty protocol quality varied: GPT-4 matched R1 (4 (2–4) vs. 4 (2–5), *p* = 0.20) and R2 (4 (2–5); *p* = 0.47) in cardiac imaging; showed no differences in neuroradiology (all 5 (1–5), *p* > 0.05); scored lower than R1 and R2 in musculoskeletal imaging (3 (2–5) vs. 4 (3–5); *p* < 0.01, and 5 (3–5); *p* < 0.001); and matched R1 (4 (1–5) vs. 2 (1–4), *p* = 0.12) as well as R2 (5 (2–5);* p* = 0.20) in oncology. GPT-4-based protocols were clinically applicable in 95% of cases, comparable to R1 (95%) and R2 (96%).

**Conclusion:**

GPT-4 generated MRI protocols with notable completeness, quality, utility, and clinical applicability, excelling in standardized subspecialties like cardiac and neuroradiology imaging while yielding lower accuracy in musculoskeletal examinations.

**Key Points:**

***Question*** *Long MRI acquisition times limit patient access, making accurate protocol selection crucial for efficient diagnostics, though it’s time-consuming and error-prone, especially for inexperienced residents.*

***Findings*** *GPT-4 generated MRI protocols of remarkable yet inconsistent quality, performing on par with an experienced resident in standardized fields, but moderately in musculoskeletal examinations.*

***Clinical relevance*** *The large language model can assist less experienced radiologists in determining detailed MRI protocols and counteract increasing workloads. The model could function as a semi-automatic tool, generating MRI protocols for radiologists’ confirmation, optimizing resource allocation, and improving diagnostics and cost-effectiveness.*

**Graphical Abstract:**

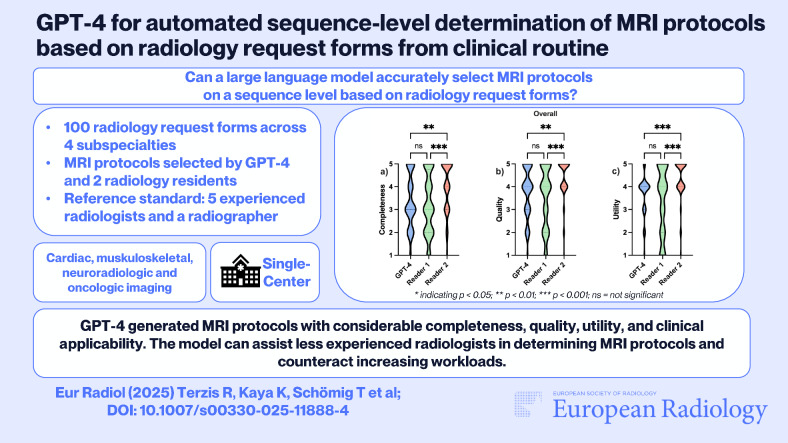

## Introduction

Magnetic resonance imaging (MRI) has established itself as the noninvasive standard of reference for various diseases of different subspecialties, e.g., myocarditis [1] or brain metastases [2]. Hence, the demand for MRI has steadily increased over the last decades, reaching 150 MRI examinations per 1000 inhabitants in Germany in 2020 [3]. Despite recent progress in scanner hardware and sequence development [4], the long acquisition times of MRI, which can range from a few minutes to over an hour in cardiac or abdominal imaging [5], limit the availability for patients. Hence, the accurate study protocol selection for MRI exams (protocoling), including the selection of appropriate sequences based on radiology request forms (RRFs), is key for effective patient throughput [6]. However, protocoling is a time-consuming task, especially for inexperienced residents, and prone to errors, leading to inefficiencies while potentially negatively impacting patient treatment [7].

Ongoing research has illustrated the benefits of employing artificial intelligence (AI) in medical decision-making, particularly in radiology, with a primary focus on the processing of imaging data [8–13]. Additionally, several approaches have been used for automated protocol determination in MRI exams, particularly leveraging machine learning and AI [14]. In this context, especially gradient boosting machine models have already achieved promising results, reaching accuracies of up to 95% for protocol selection in neuroradiology [15]. However, these models are limited by their reliance on large datasets of prior MRI protocols, typically derived from a single center [14]. Since MRI protocols are frequently adjusted to prevent protocol creep [16], align with updated guidelines, or accommodate scanner software updates, a robust automated protocoling system must be able to adapt to these changes.

While radiology and medical reports traditionally capture all essential information in natural language and text format, recent advances in large language models (LLMs) have opened up new possibilities for processing medical information within their internal knowledge base [17, 18]. One such autoregressive language model is the commercially available, Generative Pretrained Transformer fourth-generation model (GPT-4, OpenAI). Recognized for its ability to process textual data, GPT-4 represents a milestone in the field of Natural Language Processing, which employs machine learning to reveal the structure and meaning of text [19]. The model has been trained on a vast amount of text data sourced from the internet and has been refined for various linguistic tasks, including text completion, translation, and knowledge-based question-answering [20, 21]. In experimental settings, GPT-3 and -4 models have demonstrated impressive results in executing tasks typically assigned to medical professionals, demonstrating their capacity to provide logical and informational context processing [22, 23] and summarizing the complex course of patients’ disease, e.g., using information from MRI reports of glioblastoma patients [13]. Given its known ability to analyze and integrate text-based information, such models could aid in the protocoling of MRI examinations.

Hence, the objective of our study was to assess GPT-4’s performance for accurate study protocol selection on a sequence-level compared to radiology residents for different subspecialties using RRFs from clinical routine.

## Materials and methods

### Ethics

This study received ethical approval (file number 24-1001-retro), and informed consent was waived due to the retrospective design. No patient-identifying information was provided to the AI.

### Data acquisition and processing

Search criteria for this study were as follows:

1. In- or outpatient RRFs for MRI provided to the Department of Radiology for the following subspecialties: cardiac, neuroradiology (head and neck), musculoskeletal, and oncology (abdomen).
2. RRFs had to include detailed information about the patient’s medical history and a specific clinical question.
3. Patient age ≥ 18 years.

A board-certified radiologist not participating in the subsequent evaluation of MRI protocols randomly selected 25 patients from each category undergoing MRI examinations at our institution between September 2023 and February 2024, adhering to all previously mentioned inclusion criteria. During this process, a total of 17 RRFs were excluded, 15 due to insufficient information regarding the patient’s clinical history and 2 because the patients were under 18 years of age. As a result, 100 patient cases were included in the study out of a total of 117 randomly assessed RRFs. MRI examinations were performed using different systems (Philips Achieva, Ingenia, Ingenia Elition X, Intera (Philips Healthcare)) at 1.5 and 3.0 T.

All RRFs were submitted through our in-house radiology information system (Orbis-RIS version 08.42, Dedalus Healthcare System Group DACH) in the German language. Each request included the medical history and a specific clinical question, as detailed in the RRF by the referring department, in compliance with our internal standards. This information was then provided to GPT-4 (OpenAI) in text format, with a request for a detailed MRI protocol. The identical information was also provided to two radiology residents.

### GPT-4

GPT-4 was accessed through OpenAI’s web interface platform, ChatGPT (https://chat.openai.com/), between March and July 2024. Thus, prompt engineering was initiated using GPT-4 version *gpt-4-0125-preview* and finalized with *gpt-4-turbo-2024-04-09*. All RRF processing was performed using *gpt-4-turbo-2024-04-09*. Each text dataset was copied individually into a separate chat for analysis. GPT-4 evaluated each dataset using zero-shot prompting.

### Human readers

To compare GPT-4’s performance with human readers, the datasets were reviewed independently by two radiology residents with 2 years (J-P.J.; R1) and 5 years (T.S.; R2) of MRI experience. The evaluations were conducted without a specific time limit.

### Prompting

The prompt for the request to GPT-4 and human readers stated as follows:

*“Based on the medical history and the specific clinical question, provide a detailed MRI protocol to reliably exclude or secure the suspected diagnosis.”*

Radiology request forms from four patients, one of each subspeciality, were utilized by non-evaluating contributors (R.T., K.K.) to refine the prompt through 12 iterations. Each patient’s case was approached with zero-shot prompting, and the four RRFs were separately entered into an input field on the ChatGPT web interface for processing. Subsequently, prompt consistency was evaluated by processing each of the four RRFs five times to confirm stability. The four patient cases were randomly selected for each subspecialty in addition to the 100 total cases and were excluded from the final test set.

### Reference standard

Five board-certified radiologists with 9 (A.I.), 8 (L.P.), 8 (S.L.), 8 (C.G.), and 7 (J.K.) years of experience in MRI reviewed and evaluated the text-based MRI protocol recommendations generated by GPT-4 and the two radiology residents and rated them in consensus according to multiple criteria as stated below. Categories I–III) were assessed using 5-point scales. Additionally, readers were instructed to provide specific comments on the strengths and weaknesses of the protocols, including the number of unnecessary and omitted MRI sequences. Readers were unaware whether GPT-4 or the human readers generated the protocols.

(I) Overall completeness of the protocol, especially regarding the inclusion of all needed sequences in sufficient detail. Irrelevant errors are defined as errors that do not compromise the completeness of a sequence-level protocol.

1 (completely incorrect)

2 (relevant errors/omissions)

3 (multiple irrelevant errors/omissions)

4 (single irrelevant errors/omissions)

5 (no errors/omissions)

(II) Overall quality assessment of the protocols, including accuracy, consistency, and diagnostic reliability within established recommendations. Irrelevant errors are defined as errors that do not affect the diagnostic accuracy and reliability of the MRI protocol.

1 (completely incorrect)

2 (relevant errors/omissions)

3 (multiple irrelevant errors/omissions)

4 (single irrelevant errors/omissions)

5 (no errors/omissions)

(III) Assessment of the protocol’s utility in a clinical setting, considering its efficiency and specificity for the patient’s examination. An advantage is achieved when the protocol is tailored to the clinical question and remains within the essential set of sequences needed to confirm or rule out the suspected diagnosis. Negative consequences include the misallocation of MRI scan time and the disruption of clinical workflow.

1 (negative consequences)

2 (no advantage is present)

3 (advantage is minor)

4 (advantage is substantial)

5 (advantage is highly beneficial)

(IV) The GPT-4 generated protocol is presented in a logically semantic correct manner (yes/no).

Further assessment of results in category IV was conducted solely for the LLM to evaluate its ability to generate comprehensive textual output. As both radiology residents were native German speakers, their protocols were rated as being logically and semantically correct across all 100 cases.

To provide a robust reference standard and address potential biases, readers were advised to adhere to the following MRI protocol recommendations from internationally recognized radiological societies. For cardiac imaging, the standardized cardiovascular resonance imaging protocols (2020 update) of the Society for Cardiovascular Magnetic Resonance (SCMR) were employed [24]. Neuroradiological imaging adhered to the ACR-ASNR-SPR Practice Parameter for the performance of MRI of the head and neck and of the brain [25, 26] and for the performance and interpretation of MRI of the brain [26]. Musculoskeletal imaging followed the scan recommendations of the European Society of Musculoskeletal Radiology, tailored to the specific region examined, including MRI of the spine, knee, shoulder, and pelvis [27–30]. Oncology imaging protocols were applied according to the disease type, with specific guidelines for pancreatic adenocarcinoma [31], rectal cancer [32], and hepatobiliary carcinoma [33] provided by the National Comprehensive Cancer Network (NCCN).

Furthermore, the lead MRI radiographer (seven years of experience) rated the protocols’ clinical applicability using a binomial yes/no question. Clinical applicability was ensured if the radiographer could independently construct a protocol using the hardware-specific, pre-installed sequences available on each of the institution’s seven MRI scanners. It was crucial that no additional consultation with a radiologist was required for protocol implementation.

### Statistical analysis

GraphPad Prism version 9.0.1 for Mac OS X (GraphPad Software) was used for all statistical analyses. Friedman tests were conducted to evaluate differences in completeness, quality, utility, as well as the number of omitted and unnecessary sequences among GPT-4, Reader 1, and Reader 2. As post hoc tests, Dunn’s multiple comparison tests were performed. Relevance and logicality of the protocols suggested by GPT-4 were analyzed using two-tailed binomial tests. Completeness, quality, utility, and the number of omitted and unnecessary sequences are reported as median and range, whereas demographic variables are reported as mean and standard deviation. A *p*-value below 0.05 was considered statistically significant. A priori sample size calculation was performed using G*power 3.1.9.7. A minimum number of 24 participants is needed to detect a medium-sized effect of 0.6 with an alpha = 0.05 and a power of 0.8.

## Results

### Baseline characteristics

Demographic data were collected and analyzed for a total of 100 patients (*n* = 46 female, *n* = 54 male). The selected RRFs included both in- (*n* = 62) and outpatients (*n* = 38). The average age of the participants was 46.5 ± 20.3 years (range, 18-86 years).

### Overall evaluation of the generated MRI protocols

Overall, protocols determined by GPT-4 yielded a score of 3 (1–5) for completeness, of 4 (1–5) for quality, and of 4 (1–5) for utility. These results were comparable to R1, which yielded scores of 3 (1–5) for completeness, 4 (1–5) for quality, and 4 (1–5) for utility (each *p* > 0.05). However, GPT-4’s performance was inferior to the second more experienced resident (R2), who scored 4 (1–5) for completeness, 5 (1–5) for quality (both *p* < 0.01), and 5 (1–5) for utility (*p* < 0.001). In terms of the number of sequences required for optimal protocoling, both GPT-4 (1 (0–5)) and R1 (1 (0–6)) had a median of one missing necessary sequence, underperforming relative to R2 (0 (0–4); *p* = 0.04 and 0.03, respectively). A similar pattern was observed in the inclusion of unnecessary sequences, with R2 (0 (0–4)) significantly outperforming both GPT-4 (1 (0–5); *p* < 0.01) and R1 (1 (0–4); *p *< 0.01). Overall, the protocols generated by the LLM were logically and semantically correct in 80% of the cases.

**Fig. 1:**
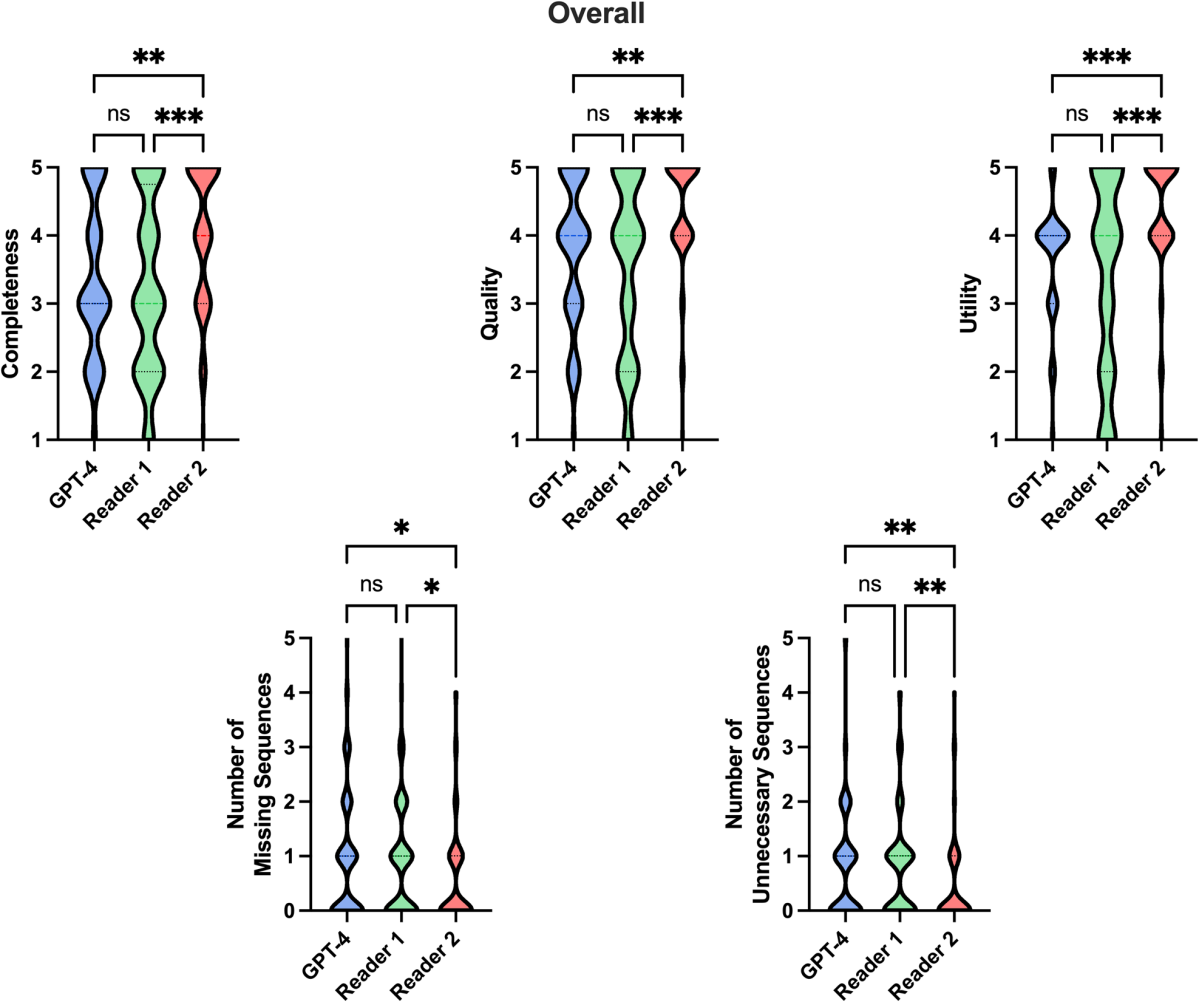
Visualization of overall performance. No significant differences were observed between GPT-4 and reader 1 in overall completeness, quality, utility, and number of sequences, whereas reader 2 yielded superior results. Reader 1 = two years of experience. Reader 2 = five years of experience. * indicating *p* < 0.05; **p < 0.01; ***p < 0.001; ns = not significant

Table 1 lists the overall performance of GPT-4 and human readers, while Fig. 1 provides a graphical visualization. The frequency of mode occurrences across the three evaluated categories for the readers and GPT-4, as determined by expert consensus on a 5-point Likert scale, can be found in Table 2. The findings related to missing and unnecessary sequences within the generated MRI protocols are presented in Table 3.

**Table 1:** Summary of median values and ranges for the expert consensus ratings of the proposed MRI protocols

				*p*-value	*p*-value	*p*-value	*p*-value
Overall	GPT-4	Reader 1	Reader 2	Friedman test	GPT-4 vs. R1	GPT-4 vs. R2	R1 vs. R2
Completeness	3 (1–5)	3 (1–5)	4 (1–5)	< 0.01	> 0.99	< 0.01	< 0.01
Quality	4 (1–5)	4 (1–5)	5 (1–5)	< 0.01	> 0.99	< 0.01	< 0.01
Utility	4 (1–5)	4 (1–5)	5 (1–5)	< 0.01	> 0.99	< 0.01	< 0.01
Cardiac							
Completeness	3 (2–4)	3 (1–3)	3 (2–5)	< 0.01	0.47	0.47	0.01
Quality	4 (2–4)	3 (1–4)	4 (2–5)	< 0.01	0.20	0.47	< 0.01
Utility	4 (2–5)	3 (1–4)	4 (1–5)	< 0.01	0.07	0.97	< 0.01
Musculoskeletal							
Completeness	2 (1–4)	3 (2–5)	4 (2–5)	< 0.01	< 0.01	< 0.01	> 0.99
Quality	3 (2–5)	4 (3–5)	5 (3–5)	< 0.01	< 0.01	< 0.01	> 0.99
Utility	3 (2–5)	4 (2–5)	5 (2–5)	< 0.01	0.01	< 0.01	> 0.99
Neuroradiology							
Completeness	5 (1–5)	5 (1–5)	5 (1–5)	0.44	> 0.99	> 0.99	> 0.99
Quality	5 (1–5)	5 (1–5)	5 (1–5)	0.16	0.97	> 0.99	> 0.99
Utility	4 (1–4)	5 (1–5)	5 (1–5)	< 0.01	0.03	< 0.01	> 0.99
Oncology							
Completeness	4 (1–5)	2 (1–4)	5 (2–5)	< 0.01	0.12	0.20	< 0.01
Quality	4 (1–5)	2 (1–4)	5 (2–5)	< 0.01	0.12	0.20	< 0.01
Utility	4 (1–5)	2 (1–4)	4 (3–5)	< 0.01	0.06	0.27	< 0.01

Reader 1 = two years of experience. Reader 2 = five years of experience

The evaluations were conducted using a 5-point Likert scale, with 5 representing the highest score

**Table 2:** Frequency of mode occurrences across all three evaluated categories (I. Completeness, II. Quality, and III. Utility of the MRI protocols), which were assessed by expert consensus on a 5-point Likert scale, with 5 indicating the highest score

Overall	1	2	3	4	5	∑
GPT-4	8 (2.7%)	43 (14.3%)	72 (24%)	111 (37%)	66 (22%)	300
Reader 1	32 (10,7%)	60 (20%)	50 (16.7%)	73 (24.3%)	85 (28.3%)	300
Reader 2	4 (1.3%)	15 (5%)	29 (9.7%)	99 (33%)	153 (51%)	300
Cardiac						
GPT-4	0 (0%)	10 (13.3%)	26 (34.7%)	38 (50.7%)	1 (1.3%)	75
Reader 1	18 (24%)	12 (16%)	28 (37.3%)	17 (22.7%)	0 (0%)	75
Reader 2	1 (1.3%)	4 (5.3%)	19 (25.3%)	35 (46.7%)	16 (21.3%)	75
Musculoskeletal						
GPT-4	1 (1.3%)	23 (30.7%)	29 (38.7%)	14 (18.7%)	8 (10.7%)	75
Reader 1	0 (0%)	3 (4%)	16 (21.3%)	25 (33.3%)	31 (41.3%)	75
Reader 2	0 (0%)	3 (4%)	9 (12%)	25 (33.3%)	38 (50.7%)	75
Neuroradiology						
GPT-4	3 (4%)	2 (2.7%)	1 (1.3%)	24 (36%)	45 (60%)	75
Reader 1	6 (8%)	11 (14.7%)	0 (0%)	4 (5.3%)	54 (72%)	75
Reader 2	3 (4%)	6 (8%)	0 (0%)	4 (5.3%)	62 (82.7%)	75
Oncology						
GPT-4	4 (5.3%)	8 (10.7%)	16 (21.3%)	35 (46.7%)	12 (16%)	75
Reader 1	8 (20.7%)	34 (45.3%)	6 (8%)	27 (36%)	0 (0%)	75
Reader 2	0 (0%)	2 (2.7%)	1 (1.3%)	35 (46.7%)	37 (49.3%)	75

Reader 1 = two years of experience. Reader 2 = five years of experience

**Table 3:** Summary of median values and ranges for the number of missing and unnecessary sequences of the proposed MRI protocols

				*p*-value	*p*-value	*p*-value	*p*-value
Overall	GPT-4	Reader 1	Reader 2	Friedman test	GPT-4 vs. R1	GPT-4 vs. R2	R1 vs. R2
Number of missing sequences	1 (0–5)	1 (0–6)	0 (0–4)	< 0.01	> 0.99	0.04	0.03
Number of unnecessary sequences	1 (0–5)	1 (0–4)	0 (0–4)	< 0.01	> 0.99	< 0.01	< 0.01
Cardiac							
Number of missing sequences	0 (0–1)	0 (0–2)	0 (0–1)	0.12	> 0.99	> 0.99	0.87
Number of unnecessary sequences	0 (0–2)	1 (0–2)	0 (0–1)	< 0.01	< 0.01	> 0.99	< 0.01
Musculoskeletal							
Number of missing sequences	2 (1–4)	1 (0–3)	1 (0–3)	< 0.01	0.01	< 0.01	0.36
Number of unnecessary sequences	2 (0–3)	1 (0–2)	0 (0–3)	< 0.01	0.04	< 0.01	0.47
Neuroradiology							
Number of missing sequences	0 (0–4)	0 (0–4)	0 (0–4)	0.44	> 0.99	> 0.99	> 0.99
Number of unnecessary sequences	1 (0-5)	0 (0-4)	0 (0-4)	0.03	0.47	0.09	> 0.99
Oncology							
Number of missing sequences	1 (0–5)	1 (0–6)	1 (0–4)	< 0.01	0.27	> 0.99	0.59
Number of unnecessary sequences	0 (0–5)	0 (0–3)	0 (0–3)	0.76	> 0.99	> 0.99	> 0.99

Reader 1 = two years of experience. Reader 2 = five years of experience

From the radiographer’s perspective, GPT-4-based protocols were clinically applicable in 95% of cases, which was comparable to R1’s applicability rate of 95% and R2’s rate of 96%.

### Evaluation of the generated MRI protocols for different subspecialties

**Fig. 2:**
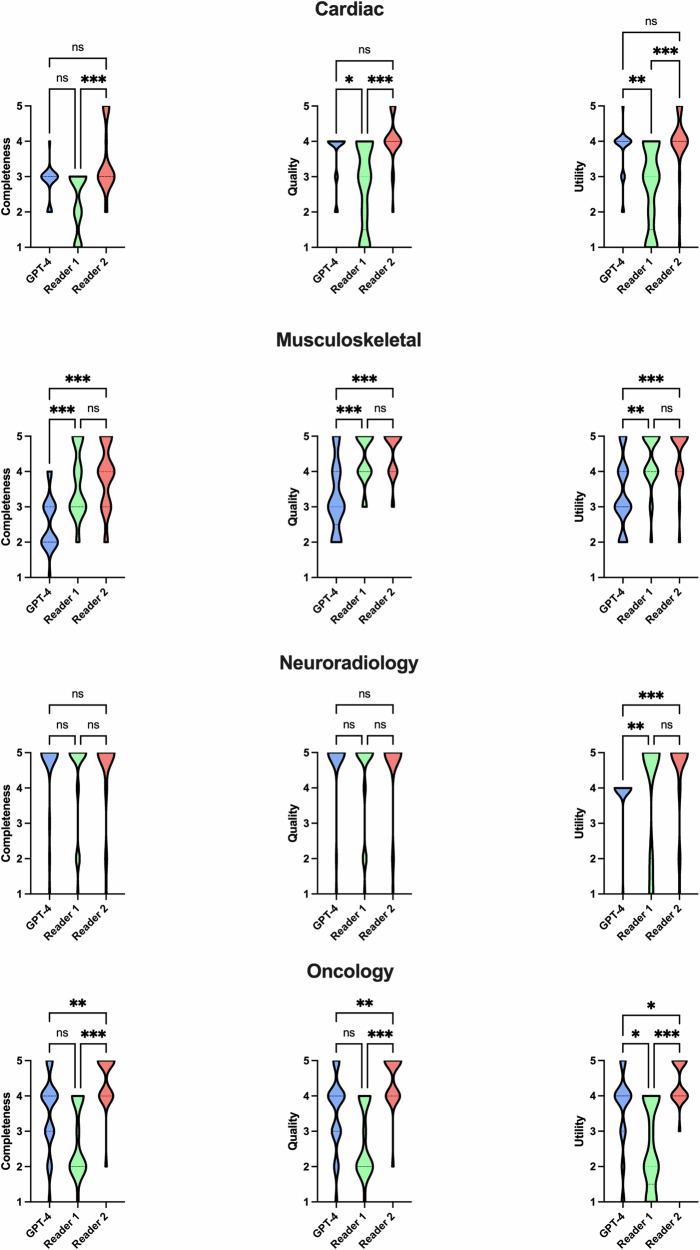
Visualization of performance for the different subspecialities. Regarding the ratings of the protocols in the different subspecialties, GPT-4 performed significantly better than reader 1 and matched reader 2 in cardiac imaging, while neuroradiology protocols generated by GPT-4 and human readers did not differ significantly. Both residents outperformed GPT-4 in musculoskeletal imaging, while GPT-4 showed slightly higher performance than reader 1 in oncologic imaging. Reader 2 demonstrated the best overall performance (*p* < 0.001). Reader 1 = two years of experience. Reader 2 = five years of experience. * indicating *p* < 0.05; **p < 0.01; ***p < 0.001; ns = not significant

Table 1 lists the overall performance of GPT-4 and human readers for different subspecialties, while Fig. 2 provides a graphical visualization.

#### Cardiac

When evaluating the protocol quality for different subspecialties, GPT-4 demonstrated a score of 4 (2–4) in cardiac imaging, which was comparable to R1’s score of 4 (2–5) (*p* = 0.20) and to R2’s score of 5 (1–5) (*p* = 047). Concordant with quality ratings, there was no significant difference in completeness between GPT-4-generated protocols, which received a score of 3 (2–4), and R1 protocols, which scored 3 (1–3) (*p* = 0.47). Imaging protocols proposed by GPT-4 (4 (2–5)) were rated to be as useful as those of R1 (3 (1–4); *p* = 0.7), also matching the performance of R2 (4 (1–5); *p* = 0.97).

Regarding the sequence-level protocols generated by GPT-4, the most frequent issue was the omission of the required views for the acquisition of sequences. This occurred in 24% of cases (6/25) and affected LGE, T1- and T2-mapping, as well as perfusion sequences.

#### Musculoskeletal

In musculoskeletal imaging, GPT-4 yielded lower results in protocol quality with a score of 3 (2–5), compared to R1’s 4 (3–5) and R2’s 5 (3–5), both showing significant differences (*p* > 0.01 and *p* < 0.001, respectively). Similarly, the scores for completeness and utility of GPT-4 protocols (2 (1–4) and 3 (2–5), respectively) were significantly lower than those of the two readers (R1: 3 (2–5) and 4 (2–5), respectively; R2: 4 (2–5) and 5 (2–5), respectively; all *p* < 0.05). No significant differences were observed between the protocols of the two raters.

GPT-4 primarily encountered difficulties with the application of proton-density spectral attenuated inversion recovery (PD-SPAIR) sequences, particularly in knee imaging. In some instances, it did not specify whether a fat-saturated T2-weighted sequence or a PD-SPAIR sequence should be preferred. Further, GPT-4 suggested including the PD-SPAIR sequence exclusively in the coronal plane instead of all three planes. As this contradicts the scan recommendations of the European Society of Musculoskeletal Radiology, it led to poor results in 20% of cases (5/25).

#### Neuroradiology

In neuroradiology, no significant difference was observed between GPT-4 and both human readers regarding protocol completeness and assessment of quality, with all scoring 5 (1–5) (each *p* > 0.05), respectively. Concerning protocol utility, the human readers slightly outperformed GPT-4 (4 (1–4)), achieving scores of 5 (1–5) for R1 (*p* = 0.03) and 5 (1–5) for R2 (*p* < 0.01). The slightly lower scores for GPT-4 in utility are attributed to the inclusion of sequences that were not explicitly necessary. For example, an additional T2-weighted sequence was suggested in stroke protocols, despite a 3D fluid-attenuated inversion recovery (FLAIR) sequence already being recommended. As a result, specificity was not optimal, and scan time could have been further reduced. This issue occurred in 28% of cases (7/25).

#### Oncology

For oncology, GPT-4 showed a slightly, but not significantly higher performance in protocol completeness and quality with a score of 4 (1–5) compared to R1’s 2 (1–4) (both *p* = 0.12), respectively, and matched R2’s scores of 5 (3–5) (*p* = 0.20), respectively. GPT-4 generated protocols demonstrated a score of 4 (1–5), which was rated to be as useful as those of R1 (2 (1–4), *p* = 0.06), with R2 yielding concordant results (4 (3–5), *p* = 0.27). Regarding the sequence-level protocols generated by GPT-4, the most frequent issue was the omission of subtraction images in cases where they were indicated according to guidelines, affecting 32% of cases (8/25). Additionally, dynamic contrast imaging of the liver was missing in 8% of cases (2/25). However, dynamic contrast imaging of the liver was required in 16 out of 25 cases and was correctly implemented in 87.5% of those instances (14/16).

**Fig. 3:**
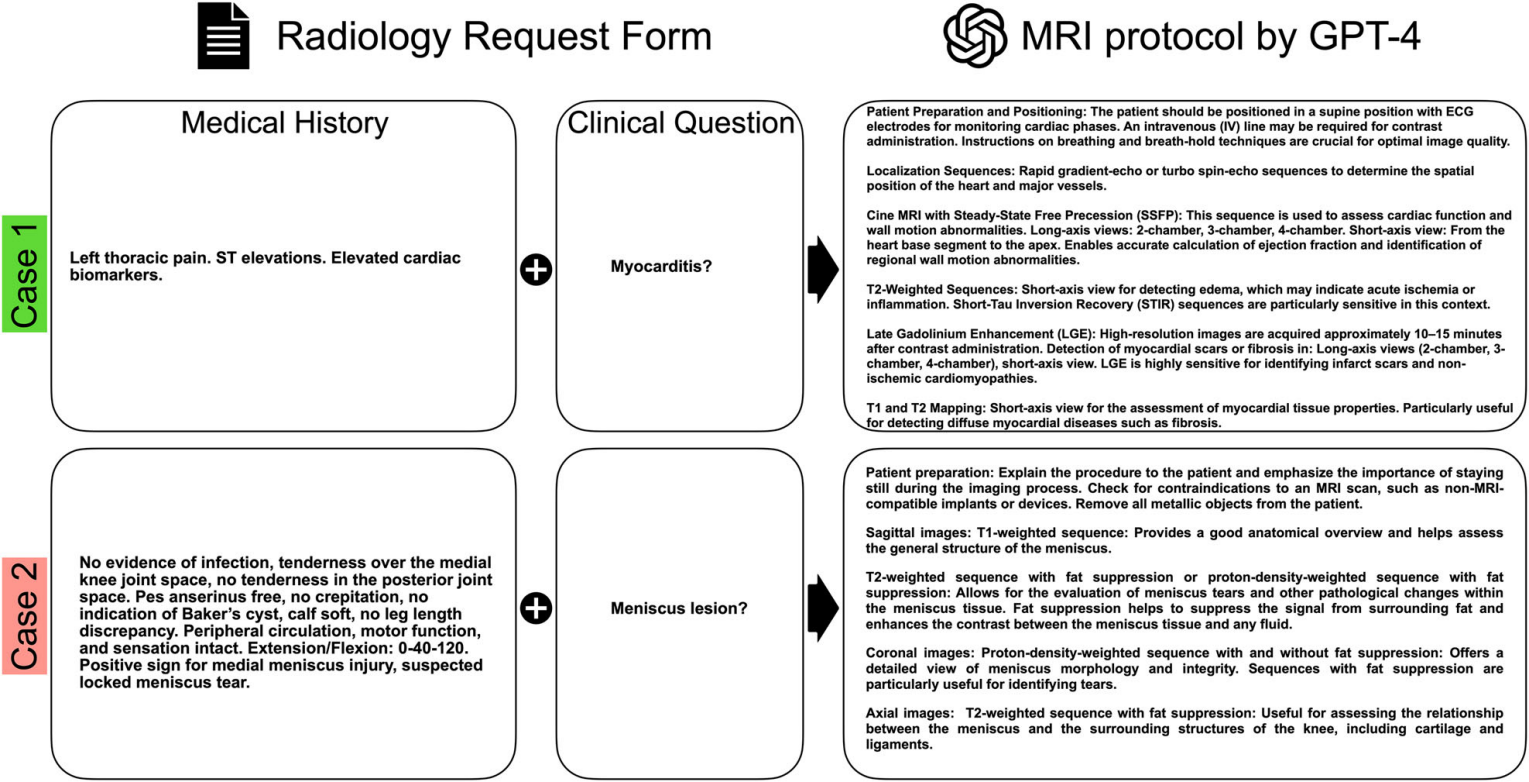
Exemplary cases of GPT-4 generated MRI protocols. Example case 1 involves a cardiac MRI examination of a 47-year-old female patient with left thoracic pain, elevated cardiac biomarkers, and suspected myocarditis. The GPT-4 generated protocol was rated particularly well by the expert consensus, yielding a score of 5 for completeness, quality, and for utility/specificity (highlighted in green). In example case 2, a 21-year-old patient was presented to our emergency department after experiencing a sudden pain in her knee while doing squats. The MRI protocol proposed by GPT-4 received poor ratings from the expert consensus, scoring 2 in completeness, quality, and utility, respectively (highlighted in red)

Figure 3 depicts exemplary cases for protocols proposed by GPT-4. Additionally, eight exemplary cases are available in the supplemental material. To this end, the information included in the RRFs as well as the MRI protocols were translated into English from the German original using GPT-4o. The expert consensus approved the translations as being concordant with the original.

## Discussion

This retrospective study investigated the potential of GPT-4, a generic LLM, for accurate study protocol selection on a sequence-level for different subspecialties using RRFs from clinical routine. Results were compared to the performance of two radiology residents with different years of experience. After evaluation of protocols by a consensus reading of five board-certified radiologists, GPT-4 generated MRI protocols with sufficient completeness, quality, utility, and clinical applicability, which were comparable to a second-year resident. Further, GPT-4 and the second-year resident performed similarly in terms of missing and unnecessary sequences, while the more experienced resident showed a slightly reduced number in both categories. The model excels at highly standardized subspecialities such as cardiac imaging and neuroradiology, whereas performance in musculoskeletal imaging was moderate. In both cardiac imaging and neuroradiology, GPT-4-generated MRI protocols were comparable to the protocols of the experienced resident. These findings highlight the potential of GPT-4 to assist less experienced radiologists in protocol determination.

To the best of our knowledge, this is the first study to examine the quality and utility of MRI protocols generated by an LLM like GPT-4 on a sequence-level. Gertz et al were able to demonstrate an accuracy of 84% for GPT-4 for the determination of the appropriate radiological study (including modality, body region, the application of contrast agent, and if yes, which contrast phases) compared to an expert reader [12]. In a different study, Lee et al evaluated the accuracy and limitations of ChatGPT in answering basic knowledge questions and specialized multiple-choice questions related to MRI [34]. The model showed good performance on simple questions with an accuracy rating over 85%; however, ratings for more specialized questions regarding the choice of the right coils, site planning, etc., received moderate ratings (40%–66.7%) [34].

In the context of medical workflow optimization, our research results deliver evidence to further expand the possible implementation of GPT-4 as a clinical decision support system. Building on the findings of Gertz et al, the LLM demonstrates promise in accurately identifying MRI as the appropriate modality when indicated [12]. In the next step, the model could suggest detailed MRI sequences to secure or rule out the suspected diagnosis. With an interface to the in-house software system, RRFs could be seamlessly processed by the LLM, eliminating the need for additional steps, as recently shown in a review by Busch et al [35]. Eventually, the AI-network could be locally integrated as a semi-automatic tool, providing MRI protocols to radiologists that only need confirmation to ensure their safe application for patients. In this context, the radiologist remains essential, as GPT-4 may occasionally misinterpret RRFs and generate inappropriate protocols. Moreover, close communication with the radiographer is crucial to adjust protocols in response to incidents or incidental findings during scanning. Moreover, when incorporating GPT-4 into medical workflows as a medical software device, an application programming interface-based implementation should be prioritized to ensure more consistent and standardized prompting behavior. Notwithstanding, LLMs pose significant data privacy concerns when processing sensitive medical information on external cloud-based platforms. These risks must be addressed by solely deploying models on secure, locally hosted servers that enable strict compliance with data protection regulations. A tool accessible to both clinicians and radiologists appears to have the potential to enhance clinical workflows, streamline examination scheduling, optimize resource allocation, and improve cost-effectiveness [12, 36]. However, it is important to acknowledge that the model’s performance varies across different subspecialties. In cardiac imaging, GPT-4 generated protocols performed well, comparable to those of an experienced radiology resident. This subspecialty benefits from an internationally recognized standard for MRI protocols established by the SCMR [24]. Neuroradiology presents a similar situation, with standardized guidelines from the ACR–ASNR–SPR practice parameter for MRI of the head, neck, and brain [25, 26]. Given these more established standards, GPT-4 also demonstrated strong performance in this subspecialty. In oncologic imaging, the MRI protocols proposed by GPT-4 received slightly lower ratings compared to those in cardiac and neuroradiological imaging. Although the NCCN provides a well-defined set of protocol recommendations [31–33], various oncological societies across different continents have guidelines that may diverge from the NCCN’s, leading to less standardization and regional preferences. This lack of uniformity is even more pronounced in musculoskeletal imaging. For example, the American College of Radiology and the European Society of Skeletal Radiology each have their own established imaging guideline parameters [28–30, 37]. In the context of this study, this can be seen as an at least partial explanation for the model’s moderate performance in musculoskeletal imaging. Especially in knee imaging, many practices customize the details of each study to optimize the examination for specific clinical questions. The selection of sequences may differ due to local preferences, available equipment, or software limitations. Since this study was limited to one center, the suggested protocols only partially matched with local standards. Further evaluation in larger patient groups addressing this issue is necessary, even given that we had a heterogeneous patient cohort. Additionally, the rapid evolution of LLMs must be considered. While our findings are promising, they represent a snapshot within a swiftly advancing technological landscape, with more capable models likely to emerge in the near future.

This study’s strengths are balanced by limitations primarily inherent to the AI model itself. AI-based models like GPT-4 function as language models that supply information without the ability to fully comprehend or interpret facts [11, 23], and their characteristic linguistic patterns may, in some cases, be identifiable by blinded readers. Hence, this could represent a source of bias since protocols generated by GPT-4 may be acknowledged as such by the reference standard. Another limitation lies in the unclear origins of GPT-4’s training data, which can lead to inconsistencies and conflicting outcomes [23]. Future studies should focus on developing LLMs with built-in transparency about the sources or guidelines underlying their decisions, enabling verification and critical evaluation. Furthermore, the retrospective design and the single-center approach are limitations of this study, as the method does not fully capture real-world scenarios where RRFs vary between sites and may sometimes contain sequence recommendations. Even with a consensus reading by five MRI experts and the relatively straightforward sequence-level recommendations by internationally recognized radiological societies, the choice of reference standard presents a limitation in the study design, as each protocol was rated individually without prior definition of a reference protocol. Additionally, GPT-4 only provided protocols on a sequence level without technical details, e.g., slice thickness, field of view, or repetition time, and did not provide assistance regarding the selection of appropriate hardware, including field strength and coils. In the future, more sophisticated models specifically trained for imaging and radiology may enable to creation of a detailed protocol; however, vendor- and field-strength-specific differences must be cautiously observed. Such models, trained on large and diverse medical datasets, have the potential to reshape how medical professionals interact with AI. Despite these advancements, communication between patients, radiologists, and radiographers remains essential, as human interaction is fundamental to delivering perceptive and empathetic care. Given the risk of misinterpreting RRFs and producing inappropriate protocols, the educational standard and final authority should continue to rest with board-certified radiologists and established clinical guidelines.

In conclusion, GPT-4 generated MRI protocols with considerable completeness, quality, usefulness, and clinical relevance. Performance differed across subspecialties, with higher accuracy observed in more standardized domains, including cardiac, oncologic, and neuroradiology imaging, and comparatively lower performance in musculoskeletal examinations. The model may hold the potential to alleviate radiologists’ workloads as a supportive tool in MRI protocoling. Future research should investigate whether an LLM can identify RRFs with insufficient clinical information to further optimize workflow efficiency. Additionally, future research should investigate whether adapting GPT-4 locally by incorporating national and in-house guidelines can validate its remarkable performance in experimental settings within a multicenter approach, ensuring the generalizability of the findings.

## Supplementary information

Supplementary information

## Abbreviations

AI: Artificial intelligence
LLM: Large language model
NCCN: National Comprehensive Cancer Network
RRF: Radiology request form
SCMR: Society for Cardiovascular Magnetic Resonance
