# Supplementary material for: GPT-4 for automated sequence-level determination of MRI protocols based on radiology request forms from clinical routine
Source: Eur Radiol. 2025 Aug 8;36(2):1541–52. doi: 10.1007/s00330-025-11888-4 (PMC12953275; doi:10.1007/s00330-025-11888-4)
Supplement: Supplementary file 1 — Supplementary information [file 330_2025_11888_MOESM1_ESM.pdf]

GPT-4 for Automated Sequence-Level Determination of MRI Protocols Based on Radiology Request Forms from Clinical Routine

ELECTRONIC SUPPLEMENTARY MATERIAL

Supplementary material 1: Exemplary cases of GPT-4 generated MRI protocols.
Representative Reports of the different subspecialities

Report 1 (Oncology)

Clinical History: Metastatic breast carcinoma (osseous, pulmonary, and hepatic). Palbociclib discontinued since 08/2019 due to severe side effects (depression).

Clinical Question: Restaging of abdominal metastases.

Recommended Protocol:

- ***Axial and coronal T1- and T2-weighted sequences for the liver***
  To assess hepatic metastases. T1-weighted images before and after contrast administration are particularly useful for differentiating hepatic lesions from normal liver tissue.
- ***Diffusion-weighted imaging (DWI) for the liver***
  DWI helps identify hepatic metastases based on cellular density and assess response to treatment.
- ***High-resolution T1-weighted sequences with fat suppression after contrast administration for the spine and pelvis***
  To evaluate osseous metastases. The sequences should cover the entire spine to detect even the smallest lesions.
- ***T2-weighted sequences with fat suppression for the lungs***
  Although CT is generally preferred for assessing pulmonary metastases, T2-weighted MRI sequences with fat suppression can provide complementary information.
- ***Whole-body MRI***
  If available, whole-body MRI can be useful for evaluating both osseous and visceral metastases and determining the extent of disease spread.

Protocol complete: 1

Protocol clinically applicable by radiographer: no

Logic semantic representation of the protocol by GPT-4: no

Overall quality: 1

Overall utility and specificity of the protocol: 1
Comments: GPT has put together a strange whole-body protocol here, including GWS and lung sequence. I interpreted this request more as an abdomen MRI request.

Report 2 (Oncology)

Clinical History: Dilated bile ducts of unclear origin.

Clinical Question: Correlation requested for sonographically suspected central left-sided dilated bile ducts. Exclusion of malignant process.

Recommended Protocol:

- ***Axial and coronal T2-weighted sequences of the upper abdomen***
  These sequences are highly sensitive to fluid, making them ideal for visualizing dilated bile ducts and assessing the liver and pancreatic parenchyma structure.
- ***High-resolution 3D MRCP***
  Provides a detailed visualization of the intrahepatic and extrahepatic bile ducts and can help identify the cause of the dilation (e.g., stones, strictures, tumors).
- ***2D MRCP***
  Can be used as a complementary technique to 3D imaging for targeted views.
- ***Dynamic axial T1-weighted sequences of the liver before and after contrast administration, including subtraction***
  Important for assessing the bile duct walls and detecting pathological lesions that may cause bile duct dilation. After contrast administration, inflammatory processes, tumors, and other lesions can be better differentiated.
- ***Diffusion-weighted imaging (DWI) of the upper abdomen***
  This technique helps distinguish between benign and malignant lesions by analyzing cell density and integrity.

Protocol complete: 4

Protocol clinically applicable by radiographer: yes

Logic semantic representation of the protocol by GPT-4: yes

Overall quality: 5

Overall utility and specificity of the protocol: 5
Comments: Decent and specific protocol to answer the clinical question.

Report 3 (Heart)

Clinical History: Cardiomyopathy with left bundle branch block. Suspected coronary artery disease and myocardial infarction scars.

Clinical Question: CAD? Cardiac ischemia?

Recommended Protocol:

Patient Preparation and Positioning:

The patient should be positioned supine with ECG electrodes for cardiac phase monitoring. An intravenous (IV) line may be required for contrast administration. Patient instructions on breathing and breath-holding are crucial for image quality.

Localization Sequences:

Fast gradient-echo or turbo spin-echo sequences to determine the spatial position of the heart and major vessels.

Cine MRI with Steady-State Free Precession (SSFP):

This sequence is used to assess cardiac function and wall motion abnormalities. Both long-axis views (2-, 3-, 4-chamber) and short-axis views (from base to apex) should be acquired to accurately calculate ejection fraction and identify regional wall motion abnormalities.

T2-Weighted Sequences:

For detecting edema, which may indicate acute ischemia or inflammation. Short-Tau Inversion Recovery (STIR) sequences are particularly sensitive in this context.

Stress Perfusion Imaging:

Following administration of a gadolinium-based contrast agent, images are acquired under pharmacological stress (e.g., with adenosine) to identify areas of reduced blood flow under stress conditions. This is particularly useful for diagnosing coronary artery disease.

Late Gadolinium Enhancement (LGE):

High-resolution images are obtained approximately 10-15 minutes after contrast administration to detect myocardial scars or fibrosis. LGE is highly sensitive for identifying infarct scars and non-ischemic cardiomyopathy.

T1 and T2 Mapping:

These quantitative techniques allow for the assessment of myocardial tissue properties and are useful for detecting diffuse myocardial diseases such as fibrosis.

Flow Measurements (Phase-Contrast MRI):

Used to evaluate heart valve function and quantify blood flow and shunt volumes.

Protocol complete: 3

Protocol clinically applicable by radiographer: no

Logic semantic representation of the protocol by GPT-4: no

Overall quality: 3

Overall utility and specificity of the protocol: 2
Comments: Non-specific => Flow measurement, mapping, and T2w are not needed; LGE views are missing, perfusion is not mentioned in the short axis.

<u>Report 4 (Heart)</u>

Clinical History:

Left thoracic pain. ST elevations. Elevated cardiac biomarkers.

Clinical Question:

Myocarditis?

Recommended Protocol:

Patient Preparation and Positioning:
The patient should be positioned in a supine position with ECG electrodes for monitoring cardiac phases. An intravenous (IV) line may be required for contrast administration. Instructions on breathing and breath-hold techniques are crucial for optimal image quality.

Localization Sequences:
Rapid gradient-echo or turbo spin-echo sequences to determine the spatial position of the heart and major vessels.

Cine MRI with Steady-State Free Precession (SSFP):
This sequence is used to assess cardiac function and wall motion abnormalities.

- ***Long-axis views: 2-chamber, 3-chamber, 4-chamber.***
- ***Short-axis view: From the heart base segment to the apex.***
- ***Enables accurate calculation of ejection fraction and identification of regional wall motion abnormalities.***

T2-Weighted Sequences:

- ***Short-axis view for detecting edema, which may indicate acute ischemia or inflammation.***
- ***Short-Tau Inversion Recovery (STIR) sequences are particularly sensitive in this context.***

Late Gadolinium Enhancement (LGE):

- ***High-resolution images are acquired approximately 10–15 minutes after contrast administration.***

- ***Detection of myocardial scars or fibrosis in:***
    - ***Long-axis views (2-chamber, 3-chamber, 4-chamber).***
    - ***Short-axis view.***
- ***LGE is highly sensitive for identifying infarct scars and non-ischemic cardiomyopathies.***

T1 and T2 Mapping:

- ***Short axis view for the assessment of myocardial tissue properties.***
- ***Particularly useful for detecting diffuse myocardial diseases such as fibrosis.***

Protocol complete: 5

Protocol clinically applicable by radiographer: yes

Logic semantic representation of the protocol by GPT-4: yes

Overall quality: 5

Overall utility and specificity of the protocol: 5
Comments: Good choice of sequences to answer the clinical question.

<u>Report 5 (Neuroradiology)</u>

Clinical History: NSCLC of the right upper lung lobe. Smoking history of 25 pack-years. Status post lobectomy of the right upper lung lobe.

Clinical Question: Brain metastases?

Recommended MRI Protocol for Lung Cancer Follow-up:

1. ***T1-weighted sequences (T1w)***
    - ***Purpose: Enables assessment of general anatomy and helps identify soft tissue structures and postoperative changes.***
    - ***Implementation: Axial and coronal slices through the thorax for detailed visualization of the surgical site and remaining lung structures.***
2. ***T2-weighted sequences (T2w) with and without fat suppression***
    - ***Purpose: Identifies fluid accumulations, inflammation, and other pathologies in the thoracic region, including the detection of residual tumors or recurrences due to the increased water content of tumor tissue.***

- *Implementation: Axial and coronal slices for comprehensive examination of the thoracic region.*

3. *Diffusion-weighted imaging (DWI)*
   - *Purpose: Evaluates lesion cellularity, which can help differentiate between postoperative changes and malignant recurrences.*
   - *Implementation: Axial slices through the thorax to identify areas of restricted diffusion that may indicate malignant activity.*
4. *Dynamic contrast-enhanced sequences (DCE-MRI)*
   - *Purpose: (Optional, depending on availability and specific clinical question) Assesses vascularity and dynamic perfusion characteristics of residual tissue or potential recurrences.*
   - *Implementation: Sequences before and after the administration of a gadolinium-based contrast agent to analyze contrast uptake and washout in suspicious areas.*

Protocol complete: 1

Protocol clinically applicable by radiographer: no

Logic semantic representation of the protocol by GPT-4: no

Overall quality: 1

Overall utility and specificity of the protocol: 1
Comments: The protocol is completely wrong. A protocol for a brain MRI was requested, but instead, a protocol for a lung MRI was provided.

<u>Report 6 (Neuroradiology)</u>

Clinical History: Tendency to fall to the right and gait disturbance. Wake-up stroke scenario.

Clinical Question: Ischemia? Hemorrhage? Vascular occlusion?

Recommended Protocol:

1. *T1-weighted sequences (T1w)*
   - *Purpose: Anatomical visualization and detection of acute and chronic hemorrhages.*
   - *Implementation: Both axial and coronal slices for a comprehensive assessment of brain structures.*

2. ***T2-weighted sequences (T2w)***
    - ***Purpose:** Detection of edema and differentiation of tissue types. Chronic ischemia and certain types of hemorrhages are better visualized in T2.*
    - ***Implementation:** Axial and coronal slices for a thorough evaluation.*
3. ***Fluid-attenuated inversion recovery (FLAIR)***
    - ***Purpose:** Highly sensitive for detecting ischemia and identifying subarachnoid and intraventricular hemorrhages.*
    - ***Implementation:** Axial sequences are particularly useful for highlighting lesions against cerebrospinal fluid (CSF).*
4. ***Diffusion-weighted imaging (DWI)***
    - ***Purpose:** Gold standard for early detection of acute ischemic strokes. Allows for the identification of infarcts within minutes to hours after symptom onset.*
    - ***Implementation:** Axial slices for rapid identification of diffusion restrictions.*
5. ***Gradient-echo sequences (GRE) or susceptibility-weighted imaging (SWI)***
    - ***Purpose:** Highly sensitive for detecting microbleeds and iron deposits, which are relevant in vascular diseases or older hemorrhages.*
    - ***Implementation:** Axial slices to identify hemorrhagic residues or microhemorrhages.*
6. ***Magnetic resonance angiography (MRA)***
    - ***Purpose:** Non-invasive visualization of vessels to assess vascular occlusions, stenoses, or aneurysms.*
    - ***Implementation:** A 3D time-of-flight (TOF) MRA can be used for intracranial vessels, while contrast-enhanced MRA may be beneficial for extracranial vessels.*
7. ***Perfusion imaging (if available)***
    - ***Purpose:** Evaluation of cerebral blood flow dynamics, which can help assess the extent and severity of ischemia.*
    - ***Implementation:** Dynamic contrast-enhanced (DCE) or dynamic susceptibility-weighted (DSC) sequences.*

General Notes:

- ***Contrast agent:** If a vascular pathology is suspected, gadolinium contrast may be useful in MRA or for detailed tissue vascularity assessment.*

Protocol complete: 5

Protocol clinically applicable by radiographer: yes

Logic semantic representation of the protocol by GPT-4: yes

Overall quality: 5

Overall utility and specificity of the protocol: 4
Comments: Protocol is answering all relevant questions; it could be reduced in time by cutting T2w and T1w-Sequences for a stroke protocol.

<u>Report 7 (Muscoloskelatal)</u>

Clinical History: Status post bicycle accident. Pain on the inner side of the right knee.

Clinical Question: Trauma-related injury?

Recommended MRI Protocol:

Sagittal Views

- ***T1-weighted sequence:***
  - ***Purpose: Assessment of general knee anatomy, including bone structure and soft tissue contours.***
- ***T2-weighted sequence with fat saturation:***
  - ***Purpose: Visualization of edema and fluid, useful for identifying traumatic changes such as bone bruises or meniscal tears.***

Coronal Views

- ***Proton density-weighted sequence with and without fat saturation:***
  - ***Purpose: Detailed examination of the menisci, ligaments, and joint cartilage. These sequences help identify meniscal tears and damage to the medial collateral ligament (MCL).***

Axial Views

- ***T2-weighted sequence with fat saturation:***
  - ***Purpose: Evaluation of the patella, patellar tendon, quadriceps tendon, and the relationship between soft tissue structures and the joint capsule. Useful for detecting fluid accumulations indicative of intra-articular injury.***

Protocol complete: 2

Protocol clinically applicable by radiographer: yes

Logic semantic representation of the protocol by GPT-4: yes

Overall quality: 2

Overall utility and specificity of the protocol: 2
Comments: According to the recommendations of the ESSR and the German Society of Musculoskeletal Radiology, the PD-SPAIR sequence should be acquired in all three planes rather than solely in the coronal view. A T2-weighted sequence may be added if necessary but should not be included by default. The T1 sequence can be included in sagittal or coronal plane, correct.

<u>Report 8 (Muscoloskelatal)</u>

Clinical History: Right sided cervicobrachialgia with intermittent weakness in the right arm.

Clinical Question: Exclusion of a herniated disc (NPP).

Recommended Protocol:

Sagittal Views

- ***T1-weighted sequence:***
  - ***Purpose: Allows assessment of anatomical structures, including vertebral bodies, intervertebral discs, and the spinal canal.***
- ***T2-weighted sequence:***
  - ***Purpose: Highlights water content and pathologies; useful for identifying inflammatory processes, edema, and herniated discs (NPP).***
- ***STIR sequence (Short Tau Inversion Recovery):***
  - ***Purpose: Highly sensitive for detecting edema and inflammation in the nerve roots and spinal cord.***

Axial Views

- ***T2-weighted sequence:***
  - ***Purpose: Provides a detailed view of the nerve roots and helps differentiate between a herniated disc (NPP) and other causes of nerve compression.***
- ***T1-weighted sequence after contrast administration (if indicated):***

- ***Purpose: Contrast agent can be useful for better differentiation of inflammatory changes or tumors. However, the indication should be assessed individually, especially in patients with renal insufficiency or known contrast agent allergy.***

Protocol complete: 4

Protocol clinically applicable by radiographer: yes

Logic semantic representation of the protocol by GPT-4: yes

Overall quality: 4

Overall utility and specificity of the protocol: 5
Comments: Very specific and useful protocol. Offering the option of a T1-weighted sequence with contrast after acquiring the initial native sequences is a smart approach, particularly when a tumor turns out to be a possible differential diagnosis. Including an oblique T2 sequence would have been perfect, but not necessarily needed.

Supplementary material 2: List of all 100 included radiology request forms from clinical routine.

List of all 100 included radiology request forms from clinical routine sorted by subspecialties

Translation from German to English was performed using GPT-4o (accessed 05th June 2025). The authors approved the translations being concordant to the original.

1. **Cardiac imaging**

Radiology Request Form 1

German (Original Radiology Request Form):

Anamnese: Kardiomyopathie bei Linksschenkelblock. Verdacht auf koronare Herzkrankheit sowie Infarktnarben.

Fragestellung: KHK? Kardiale Ischämien?

English:

Medical History**:** Cardiomyopathy with left bundle branch block. Suspected coronary artery disease and myocardial scarring.

Clinical Question**:** Coronary artery disease? Cardiac ischemia?

Radiology Request Form 2

German (Original Radiology Request Form):

Anamnese: Arterielle Hypertonie. Eingeschränkte Belastbarkeit.

Fragestellung: Myokarditis? Kardiale Auffälligkeiten?

English:

Medical History**:** Arterial hypertension. Reduced exercise tolerance.

Clinical Question**:** Myocarditis? Cardiac abnormalities?

Radiology Request Form 3

German (Original Radiology Request Form):

Anamnese: Patient mit OHCA bei Vorhofflimmern nach Kardioversion einer supraventrikulären Tachykardie.

Fragestellung: Akuter Infarkt? Myokarditis?

English:

Medical History**:** Patient with OHCA (out-of-hospital cardiac arrest) due to atrial fibrillation following cardioversion of a supraventricular tachycardia.

Clinical Question**:** Acute myocardial infarction? Myocarditis?

Radiology Request Form 4

German (Original Radiology Request Form):

Anamnese: Patient mit Herzinsuffizienz unklarer Ätiologie. Begleitende Fatigue.

Fragestellung: KHK? Kardiale Ischämien?

English:

Medical History**:** Patient with heart failure of unclear etiology. Accompanying fatigue.

Clinical Question**:** Coronary artery disease? Cardiac ischemia?

Radiology Request Form 5

German (Original Radiology Request Form):

Anamnese: Hypertensive Entgleisung, Troponinämie unter Infekt mit begleitender Dyspnoe.

Fragestellung: Myokarditis?

English:

Medical History**:** Hypertensive crisis, elevated troponin levels during infection with accompanying dyspnea.

Clinical Question**:** Myocarditis?

Radiology Request Form 6

German (Original Radiology Request Form):

Anamnese: Zustand nach Out of Hospital Cardiac Arrest. Primäres Rhythmusereignis mit Kammerflimmern.

Fragestellung: Myokardinfarkt? Kardiale Auffälligkeiten?

English:

Medical History**:** Status post out-of-hospital cardiac arrest. Primary arrhythmic event with ventricular fibrillation.

Clinical Question**:** Myocardial infarction? Cardiac abnormalities?

Radiology Request Form 7

German (Original Radiology Request Form):

Anamnese: Zustand nach Out of Hospital Cardiac Arrest. Ventrikelseptumdefekt, am ehesten bei Ruptur.

Fragestellung: Infarktnarbe? Kardiale Auffälligkeiten?

English:

Medical History**:** Status post out-of-hospital cardiac arrest. Ventricular septal defect, most likely due to rupture.

Clinical Question**:** Myocardial scar? Cardiac abnormalities?

Radiology Request Form 8

German (Original Radiology Request Form):

Anamnese: Kardiomegalie. LV-Hypertrophie ohne echokardiografisch relevante LV-Obstruktion

Fragestellung: HOCM? Kardiale Auffälligkeiten?

English:

Medical History**:** Cardiomegaly. Left ventricular hypertrophy without echocardiographically relevant LV outflow obstruction.

Clinical Question**:** HOCM (Hypertrophic Obstructive Cardiomyopathy)? Cardiac abnormalities?

Radiology Request Form 9

German (Original Radiology Request Form):

Anamnese: Arterielle Hypertonie. Eingeschränkte Belastbarkeit. Adipositas per Magna. In der Echokardiographie LV-Thrombus nicht auszuschließen.

Fragestellung: LV-Thrombus? DCM? Kardiale Auffälligkeiten?

English:

Medical History: Arterial hypertension. Reduced exercise capacity. Morbid obesity. Left ventricular thrombus cannot be ruled out on echocardiography.

Clinical Question: LV thrombus? Dilated cardiomyopathy (DCM)? Cardiac abnormalities?

Radiology Request Form 10

German (Original Radiology Request Form):

Anamnese: Patient mit Verdacht auf Amyloidose. Freie kappa Leichtketten und kappa/lambda Quotient im Serum leicht erhöht, Immunfixation in Serum und Urin ohne H.a. monoklonale Gammopathie

Fragestellung: Kardiale Amyloidose?

English:

Medical History: Patient with suspected amyloidosis. Free kappa light chains and kappa/lambda ratio in serum slightly elevated; immunofixation in serum and urine without evidence of monoclonal gammopathy.

Clinical Question: Cardiac amyloidosis?

Radiology Request Form 11

German (Original Radiology Request Form):

Anamnese: Patient mit Verdacht auf koronare Herzkrankheit. Fatigue.

Fragestellung: Kardiale Ischämie?

English:

Medical History: Patient with suspected coronary artery disease. Fatigue.

Clinical Question: Cardiac ischemia?

Radiology Request Form 12

German (Original Radiology Request Form):

Anamnese: Strukturell auffälliger RV und langjährige Rhythmusstörungen.

Fragestellung: Hinweise auf Kardiomyopathie, ARVCM?

English:

Medical History: Structurally abnormal right ventricle and long-standing arrhythmias.

Clinical Question: Evidence of cardiomyopathy, ARVC (Arrhythmogenic Right Ventricular Cardiomyopathy)?

Radiology Request Form 13

German (Original Radiology Request Form):

Anamnese: Hochgradig eingeschränkte LV-Funktion

Fragestellung: Strukturelle Herzerkrankung?

English:

Medical History: Severely reduced left ventricular function.

Clinical Question: Structural heart disease?

Radiology Request Form 14

German (Original Radiology Request Form):

Anamnese: Verdacht auf Myokarditis

- seit Samstag rezidivierendes thorakales Engegefühl, Sonntag morgen thorakaler Schmerz in Ruhe mit Ausstrahlung in linken Arm

- seit 1 Woche Kopfschmerzen, Abgeschlagenheit

- Trop: 0,137, CK 114, CKMB 28

- oTTE: gute LVEF, kein PE

Fragestellung: (Peri-)Myokarditis?

English:

Medical History: Suspected myocarditis

- Recurrent chest tightness since Saturday; resting chest pain radiating to the left arm on Sunday morning

- Headaches and fatigue for one week
- Troponin: 0.137, CK: 114, CKMB: 28
- TTE: good LVEF, no pericardial effusion

Clinical Question**:** (Peri)myocarditis?

Radiology Request Form 15

German (Original Radiology Request Form):

Anamnese: Verdacht auf Non Compaction Cardiomyopathia

Fragestellung: Strukturelle Herzerkrankung?

English:

Medical History**:** Suspected noncompaction cardiomyopathy.

Clinical Question**:** Structural heart disease?

Radiology Request Form 16

German (Original Radiology Request Form):

Anamnese: Verdacht auf HCM.

Fragestellung: Strukturelle Herzerkrankung?

English:

Medical History**:** Suspected hypertrophic cardiomyopathy (HCM).

Clinical Question**:** Structural heart disease?

Radiology Request Form 17

German (Original Radiology Request Form):

Anamnese: Im CT vorgeschriebene RF an der Mitraklappe, den LVOT obstruierend, echokardiograpisch bei eingeschränkten Schallbedingungen nicht nachvollziehba

Fragestellung: Herz-Tumor, Thrombus, Degeneration an MK?

English:

Medical History**:** CT revealed a space-occupying lesion at the mitral valve, obstructing the LVOT; not clearly visualized on echocardiography due to limited acoustic windows.

Clinical Question**:** Cardiac tumor, thrombus, or mitral valve degeneration?

Radiology Request Form 18

German (Original Radiology Request Form):

Anamnese:

Aortenbogenhypoplasie mit Isthmusstenose

Milde valvuläre Aortenstenose

Z.n. Aortenbogenpatcherweiterung, Kommissurotomie und Ductusverschluss (26.05.2009, Uni Köln)

Verschluss der Arteria brachialis rechts

Z.n. cerebralen Krampfanfall beim Neugeborenen

Aktuell:

Geringgradige Aortenklappenstenose und -insuffizienz

Milde LV Hypertrophie

Fragestellung: Re-Stenose Aorta? LV-Hypertrophie?

English:

Medical History:

- Aortic arch hypoplasia with isthmus stenosis
- Mild valvular aortic stenosis
- Status post aortic arch patch enlargement, commissurotomy, and ductus closure (May 26, 2009, University of Cologne)
- Occlusion of the right brachial artery
- History of neonatal cerebral seizure

Current Findings:

- Mild aortic valve stenosis and regurgitation
- Mild left ventricular hypertrophy

Clinical Question: Re-stenosis of the aorta? LV hypertrophy?

Radiology Request Form 19

German (Original Radiology Request Form):

Anamnese: Ebstein Anomalie

Fragestellung: Größe (auch indexiert auf KÖF) und Funktion des RV? Grad Trikuspidalklappeninsuffizienz MR-morphologisch?

English:

Medical History**:** Ebstein's anomaly

Clinical Question**:** Size (including body surface area–indexed) and function of the right ventricle? Degree of tricuspid regurgitation on MR imaging?

Radiology Request Form 20

German (Original Radiology Request Form):

Anamnese: Pat. mit Atrialer Switch-Operation bei D-Transposition der großen Gefäße (TGA)

Fragestellung: Evaluation der Dimensionen des systemischen

rechten Ventrikels (Größe? Funktion? Grad der Undichtigkeit der systemischen AV-Klappe). Evaluation des subpulmonalen Ventrikels.

English:

Medical History**:** Patient with atrial switch operation for D-transposition of the great arteries (TGA)

Clinical Question**:** Evaluation of the dimensions of the systemic right ventricle (size? function? degree of regurgitation of the systemic AV valve). Evaluation of the subpulmonary ventricle.

Radiology Request Form 21

German (Original Radiology Request Form):

Anamnese: Fallot Tetralogie. 2-malige Operation (RVOT/ Pulmonalklappe)

Fragestellung: Evaluation der RV-Funktion und Dimension, Relevanz der Pulmonalklappeninsuffizienz (echokardiographisch hochgradig undicht). Vermessung des RVOT/ der Pulmonalklappenebene

English:

Medical History**:** Tetralogy of Fallot. Two previous surgeries (RVOT/pulmonary valve)

Clinical Question**:** Evaluation of right ventricular function and dimensions, assessment of the significance of pulmonary valve regurgitation (severe on echocardiography). Measurement of the RVOT and pulmonary valve level.

Radiology Request Form 22

German (Original Radiology Request Form):

Anamnese:

Pulmonalklappenstenose

Ventrikelseptumdefekt

Z. n. Pulmonalklappen-Valvuloplastie in Mainz

Z. n. VSD-Direktverschluss und Pulmonalklappenkommisurotomie 03/00 in Mainz

Z. n. diagnostischer Herzkatheteruntersuchung am 22.09.2014: Melodyklappenimplantation nicht möglich

Z. n. Pulmonalklappenersatz mittels Bioprothese und Patcherweiterung der LPA am 18.11.2014

Z. n. Stentimplantation in LPA (Palmaz Schatz XL 10 x 30 mm) am 02.11.2015

Z. n. frustranem Versuch einer Ballondilatation des Stents im Bereich der LPA am 01.09.2016

Z. n. postinterventioneller Lungenblutung mit Hämoptysen

Aktuell:

Hochgradige Insuffizienz und mittelgradige Stenose (Vmax. 3,3 m/s: Pmax 40-45 mmHg) der Bioprothese in PK-Position

Dilatierte RPA

Mittelgradige Stenose der LPA (Vmax. 3 m/s)

Erhöhter RV-Druck (TKI: 40 mmHg abschätzbar)

Fragestellung: Größe (auch indexiert) und Funktion des rechten

Ventrikels? Grad der Insuffizienz und Stenose der

Bioprothese in PK-Position MR-morphologisch?

LV-Größe und LV-Funktion? Weite der Pulmonalarterien?

Pulmonalstenosen?

English:

Medical History:

- Pulmonary valve stenosis
- Ventricular septal defect (VSD)
- Status post pulmonary valve valvuloplasty in Mainz
- Status post direct VSD closure and pulmonary valve commissurotomy in 03/2000 in Mainz
- Status post diagnostic cardiac catheterization on 22.09.2014: Melody valve implantation not feasible
- Status post pulmonary valve replacement with bioprosthesis and patch enlargement of the left pulmonary artery (LPA) on 18.11.2014
- Status post stent implantation in the LPA (Palmaz Schatz XL 10 x 30 mm) on 02.11.2015
- Status post unsuccessful attempt at balloon dilation of the stent in the LPA on 01.09.2016
- Status post post-interventional pulmonary hemorrhage with hemoptysis

Current Findings:

- Severe regurgitation and moderate stenosis (Vmax 3.3 m/s; Pmax 40–45 mmHg) of the bioprosthesis in pulmonary valve position
- Dilated right pulmonary artery (RPA)
- Moderate stenosis of the LPA (Vmax 3.0 m/s)
- Elevated right ventricular pressure (estimated tricuspid regurgitation gradient: 40 mmHg)

Clinical Question:

- Size (including indexed values) and function of the right ventricle?
- Degree of regurgitation and stenosis of the bioprosthesis in pulmonary valve position on MR imaging?
- Left ventricular size and function?
- Diameter of the pulmonary arteries?
- Pulmonary artery stenoses?

Radiology Request Form 23

German (Original Radiology Request Form):

Anamnese: Verdacht auf ein Vorhofmyxom, DD Thrombus im rechten Vorhof.

Fragestellung: Dignität?

English:

Medical History**:** Suspected atrial myxoma, differential diagnosis: thrombus in the right atrium.

Clinical Question**:** Nature of the lesion (benign vs. malignant)?

Radiology Request Form 24

German (Original Radiology Request Form):

Anamnese: Unklare Raumforderung im apikoseptalen linken Ventrikel

- ca. 0,9x0,8 cm, wandständig, hyperechogen

Hypertensive Entgleisung bei schwer-einstellbarem Hypertonus

- RR 210/120 mmHg

Hypotone Hyponatriämie

- Serum: Na+ 126 mmol/L
- fraktionelle Harnstoffexkretion 51.1 %

Vorerkrankungen:

Paroxysmales Vorhofflimmern

- OAK mit Apixaban

Fragestellung: Unklare kardiale Raumforderung, bitte um Mitbeurteilung.

English:

Medical History:
Unclear mass in the apicoseptal region of the left ventricle

- Approximately 0.9 x 0.8 cm, wall-adherent, hyperechogenic

Hypertensive crisis with difficult-to-control hypertension

- BP 210/120 mmHg

Hypotonic hyponatremia

- Serum Na^+: 126 mmol/L
- Fractional urea excretion: 51.1%

Past Medical History:
Paroxysmal atrial fibrillation

- Anticoagulation with apixaban

Clinical Question: Unclear cardiac mass—please provide further assessment.

Radiology Request Form 25

German (Original Radiology Request Form):

Anamnese: Pat stationär aktuell für LAE.

Im TTE LA-Thrombus DD Myxom.

Fragestellung: Unklare Raumforderung im linken Atrium:

Myxom? Tumor? Thrombus?

English:

Medical History**:** Patient currently hospitalized for pulmonary embolism (PE). Left atrial mass seen on TTE—differential diagnosis: thrombus vs. myxoma.

Clinical Question**:** Unclear mass in the left atrium: Myxoma? Tumor? Thrombus?

2. **Neuroradiology (head and neck)**

Radiology Request Form 1

German (Original Radiology Request Form):

Anamnese: Fallneigung nach rechts und Gangstörung. Wake-Up Konstellation.

Fragestellung: Ischämie? Blutung? Gefäßabbruch?

English:

Medical History**:** Patient currently hospitalized for pulmonary embolism (PE). Left atrial mass seen on TTE—differential diagnosis: thrombus vs. myxoma.

Clinical Question**:** Unclear mass in the left atrium: Myxoma? Tumor? Thrombus?

Radiology Request Form 2

German (Original Radiology Request Form):

Anamnese: Z.n. Polytrauma als Führer eines Kleintransporters mit Unfall durch aufprallenden LKW-Anhänger. Schweres Schädel Hirn Trauma mit multiplen Mittelgesichtsfrakturen, Unterkieferfraktur, Felsenbeinfraktur und Kalottenfraktur. Aktuell Verdacht auf Aphasie.

Fragestellung: Stroke?

English:

Medical History**:** Status post polytrauma as the driver of a light commercial vehicle involved in a collision with an impacting truck trailer. Severe traumatic brain injury with multiple midface fractures, mandibular fracture, petrous bone fracture, and skullcap fracture. Currently, suspected aphasia.

Clinical Question**:** Stroke?

Radiology Request Form 3

German (Original Radiology Request Form):

Anamnese: Superfiziell spreitendes malignes Melanom, TD 1,2 mm, Oberschenkel rechts.

Fragestellung: z. A. Filiae

English:

Medical History**:** Superficially spreading malignant melanoma, tumor thickness 1.2 mm, right thigh.

Clinical Question**:** Suspicion of metastases.

Radiology Request Form 4

German (Original Radiology Request Form):

Anamnese: Patientin mit rezidivierender depressiver Störung und teilweise schwerer depressvier Episode. Erster stationärer Aufenthalt. Antriebsarmut, gedrückte Stimmung, Grübeln, Angstzustände und innere Wut. Passive lebensmüde Gedanken.

Fragestellung: Ausschluss hirnorganische Ursache der Depression.

English:

Medical History**:** Patient with recurrent depressive disorder and partially severe depressive episode. First inpatient stay. Symptoms include lack of drive, low mood, rumination, anxiety, and inner anger. Passive suicidal thoughts.

Clinical Question**:** Rule out an organic brain cause of the depression.

Radiology Request Form 5

German (Original Radiology Request Form):

Anamnese: Z.n. Akustikusneurinom rechts

Fragestellung: Rezidiv?

English:

Medical History**:** Status post right-sided acoustic neuroma.

Clinical Question**:** Recurrence?

Radiology Request Form 6

German (Original Radiology Request Form):

Anamnese: Verdacht auf Prolaktinom

Fragestellung: Hinweis auf ein Hypophysenadenom bei milder

Hyperprolaktinämie

English:

Medical History**:** Suspected prolactinoma

Clinical Question**:** Evidence of a pituitary adenoma in the context of mild hyperprolactinemia?

Radiology Request Form 7

German (Original Radiology Request Form):

Anamnese: Z.n. Resektion einer links temporalen Metastase

Fragestellung: Nachblutung? Schwellung? Resttumor?

English:

Medical History**:** Status post resection of a left temporal metastasis

Clinical Question**:** Postoperative hemorrhage? Swelling? Residual tumor?

Radiology Request Form 8

German (Original Radiology Request Form):

Anamnese: Hemihypästhesie links und Anarthrie für wenige Minuten

Fragestellung: Ischämie, Gefäßpathologie?

English:

Medical History**:** Left-sided hemihypesthesia and anarthria lasting a few minutes.

Clinical Question**:** Ischemia? Vascular pathology?

Radiology Request Form 9

German (Original Radiology Request Form):

Anamnese: Kopfschmerz, rezidivierende Kopfschmerzen, Mutter wohl Hirnbasisaneurysmablutung

Fragestellung: Zum Ausschluss Aneurysma sowie einer

Blutung.

English:

Medical History**:** Headache, recurrent headaches; mother Radiology Request Formedly had a basal cerebral aneurysm rupture.

Clinical Question**:** To rule out aneurysm and possible bleeding.

Radiology Request Form 10

German (Original Radiology Request Form):

Anamnese: Aneurysmatische SAB H&H 3 Fisher Grad III WFNS Grad II

- Acom-Aneurysma (gecoilt am 10.03)

Z.n. Anlage VP Shunt 22.04.2023

Fragestellung: ACOM-Aneurysma versorgt. Erbitte AngioMRT des Schädels

mit TOF gemäß Empfehlung des NVB.

English:

Medical History: Aneurysmal subarachnoid hemorrhage (SAH), Hunt & Hess grade 3, Fisher grade III, WFNS grade II

- ACom aneurysm (coiled on 10.03)
- Status post ventriculoperitoneal (VP) shunt placement on 22.04.2023

Clinical Question: ACom aneurysm treated. Requesting cranial MR angiography with TOF as recommended by the neurovascular board (NVB).

Radiology Request Form 11

German (Original Radiology Request Form):

Anamnese: Coiling eines

Inzidentelles paraophtalmisches Aneurysma der linken A. carotis interna (ACI) (ca. 3,5 x 3 mm)

- intraarterielle Lyse mit 10mg

- intravenöse Lyse mit 58 mg rtPA

Clipping eines bei inzidentellem Mediabifurkationsaneurysma links (ca. 1 x 2 mm) am 02.11.2021

- Akut-symptomatische Anfälle 11/2021

Inzidentelles Mediabifurkationsaneurysma rechtsseitig in statu nascendi

Fragestellung: Verlaufskontrolle ACI-Aneurysma links nach Coiling

English:

Medical History:
Coiling of an incidental paraophthalmic aneurysm of the left internal carotid artery (ICA) (approximately 3.5 x 3 mm)

- Intra-arterial thrombolysis with 10 mg
- Intravenous thrombolysis with 58 mg rtPA

Clipping of an incidental left middle cerebral artery bifurcation aneurysm (approximately 1 x 2 mm) on 02.11.2021

- Acute symptomatic seizures in 11/2021
  Incidental right-sided middle cerebral artery bifurcation aneurysm in statu nascendi

Clinical Question: Follow-up of left ICA aneurysm post coiling.

Radiology Request Form 12

German (Original Radiology Request Form):

Anamnese: Mediabifurkationsaneurysma links nach Coiling und Stentversorgung.

Fragestellung: Verlaufskontrolle.

English:

Medical History: Left middle cerebral artery bifurcation aneurysm treated with coiling and stent placement.

Clinical Question: Follow-up evaluation.

Radiology Request Form 13

German (Original Radiology Request Form):

Anamnese: Zunehmende Oculomotorius-Parese links (seit 6 Monate Befund deutlich zunehmend). Geringe Oculomotorius-Parese schon seit 2019 bekannt

Fragestellung: Zum Ausschluss Tumor, DD Oculomotorius-Neurinom

English:

Medical History**:** Progressive left oculomotor nerve palsy (marked progression over the past 6 months). Mild oculomotor palsy known since 2019.

Clinical Question**:** To rule out tumor, differential diagnosis: oculomotor schwannoma.

Radiology Request Form 14

German (Original Radiology Request Form):

Anamnese: Psychose, bisher keine Bildgebung erfolgt

Fragestellung: Organische Genese?

English:

Medical History**:** Psychosis; no imaging performed to date.

Clinical Question**:** Organic cause?

Radiology Request Form 15

German (Original Radiology Request Form):

Anamnese: NSCLC, pulmonales Adenokarzinom des rechten Lungenoberlappens. Nikotinabusus 25 pack years. Hypothyreose. Arterielle Hypertonie. Lobektomie des rechten Lungenoberlappens.

Fragestellung: Nachsorge.

English:

Medical History**:** NSCLC, pulmonary adenocarcinoma of the right upper lobe. History of nicotine abuse (25 pack years). Hypothyroidism. Arterial hypertension. Lobectomy of the right upper lobe

Clinical Question**:** Follow-up.

Radiology Request Form 16

German (Original Radiology Request Form):

Anamnese: Rezidiv eines non-Germinom der Pinealisloge. Zustand nach Chemotherapie und Radiatio. Aktuell Nachsorge

Fragestellung: Konstanter Befund? Progress? Neue Läsionen?

English:

Medical History**:** Recurrence of a non-germinoma in the pineal region. Status post chemotherapy and radiation therapy. Currently in follow-up.

Clinical Question**:** Stable findings? Progression? New lesions?

Radiology Request Form 17

German (Original Radiology Request Form):

Anamnese: Verdacht auf vertebrobalisäre TIA mit passagerem Schwindel und Synkope.

Fragestellung: Zum Ausschluss stumme Ischämien.

English:

Medical History**:** Suspected vertebrobasilar TIA with transient dizziness and syncope.

Clinical Question**:** To rule out silent ischemia.

Radiology Request Form 18

German (Original Radiology Request Form):

Anamnese: Multiple neurologische Ausfälle seit ca. 2 Monaten, nochmal Verschlechterung seit 2 Tagen. Ataktisches Gangbild, 2x Sturz in letzter Woche. Dysästhesie/Pallhypästhesie Hände und Füße.

Fragestellung: Entzündliche Läsionen, Raumforderung?

English:

Medical History**:** Multiple neurological deficits for approximately 2 months, with further deterioration over the past 2 days. Ataxic gait, two falls in the past week. Dysesthesia and impaired vibration sense in hands and feet.

Clinical Question**:** Inflammatory lesions? Space-occupying lesion?

Radiology Request Form 19

German (Original Radiology Request Form):

Anamnese: Trigeminusneuralgie links. Vor einigen Monaten Gleichgewichtsstörungen. Bekannte inadäquate Sinustachykardie.

Fragestellung: Ausschluss einer Multiplen Sklerose.

English:

Medical History**:** Left-sided trigeminal neuralgia. Balance disturbances a few months ago. Known inappropriate sinus tachycardia.

Clinical Question**:** Rule out multiple sclerosis.

Radiology Request Form 20

German (Original Radiology Request Form):

Anamnese: Last seen well 08:00 Uhr. Nun Hemiparese links. Orale Antikoagulation seit gestern pausiert.

Fragestellung: Akute Ischämien? Blutung?

English:

Medical History**:** Last seen well at 08:00. Now presenting with left-sided hemiparesis. Oral anticoagulation paused since yesterday.

Clinical Question**:** Acute ischemia? Hemorrhage?

Radiology Request Form 21

German (Original Radiology Request Form):

Anamnese: Verdacht auf linkshemisphärische Ischämie mit Wesensänderung, Desorientierung und leichtgradiger brachiofazial betonter Hemiparese rechts

Fragestellung: Hinweise für Ischämien/Gefäßabbruch/Blutung?

English:

Medical History**:** Suspected left hemispheric ischemia with personality changes, disorientation, and mild right-sided hemiparesis, more pronounced in the arm and face.

Clinical Question**:** Evidence of ischemia, vessel occlusion, or hemorrhage?

Radiology Request Form 22

German (Original Radiology Request Form):

Anamnese: Patient mit dreimaliger Amaurosis fugax

Fragestellung: Frage nach stummen Ischämien?

English:

Medical History**:** Patient with three episodes of amaurosis fugax.

Clinical Question**:** Evidence of silent ischemia?

Radiology Request Form 23

German (Original Radiology Request Form):

Anamnese: Patient mit homonymer Hemianopsie nach rechts seit gestern

Fragestellung: Ischämie? Gefäßpathologien?

English:

Medical History**:** Patient with right homonymous hemianopsia since yesterday.

Clinical Question**:** Ischemia? Vascular pathologies?

Radiology Request Form 24

German (Original Radiology Request Form):

Anamnese: Kopfschmerz, Schwindel, Übelkeit, Erbrechen, plötzlich aufgetreten gegen 21:30

Fragestellung: Ischämie? Blutung? Gefäßabbruch?

English:

Medical History**:** Headache, dizziness, nausea, and vomiting, with sudden onset around 21:30.

Clinical Question**:** Ischemia? Hemorrhage? Vessel occlusion?

Radiology Request Form 25

German (Original Radiology Request Form):

Anamnese: Verdacht auf Arteriitis temporalis, Diplopie und Defizit bei Aufwärtsblick. Unklare Dauer.

Fragestellung: Ischämie? Blutung? Gefäßabbruch?

English:

Medical History**:** Suspected temporal arteritis, diplopia, and upward gaze palsy. Duration unclear.

Clinical Question**:** Ischemia? Hemorrhage? Vessel occlusion?

3. Oncologic imaging (abdomen)

Radiology Request Form 1

German (Original Radiology Request Form):

Anamnese: Korrelat zu auswärtig erhöhten CA-19-9-Werten? Im hiesigen aktuellen Labor normale CA 19-9-Werte.

Fragestellung: Malignom?

English:

Medical History**:** Correlation with elevated CA 19-9 levels Radiology Request Formed externally? CA 19-9 levels are within the normal range in current local lab results.

Clinical Question**:** Malignancy?

Radiology Request Form 2

German (Original Radiology Request Form):

Anamnese: Abklärung genetische Disposition Pankreas. Patient derzeit keine Beschwerden. Untersuchung als Vorsoge bei familiärer Disposition eines Pankreaskarzinoms (Vater, Schwester) gedacht.

Fragestellung: Malignom?

English:

Medical History**:** Evaluation of genetic predisposition for pancreatic disease. Patient currently asymptomatic. Examination intended as a preventive measure due to family history of pancreatic cancer (father, sister).

Clinical Question**:** Malignancy?

Radiology Request Form 3

German (Original Radiology Request Form):

Anamnese: Pankreasschwanzzyste DD muzinöse Neoplasie DD BD-IPMN DD Psuedozyste

Fragestellung: Bitte um Mitbeurteilung via MRCP.

English:

Medical History**:** Pancreatic tail cyst – differential diagnosis includes mucinous neoplasm, branch-duct IPMN, or pseudocyst.

Clinical Question**:** Request for further evaluation via MRCP.

Radiology Request Form 4

German (Original Radiology Request Form):

Anamnese: Pankreaskorpus-CA geplant zur totalen Pankreatektomie

Fragestellung: Bitte Verlaufsdarstellung des Befundes.

English:

Medical History**:** Pancreatic body carcinoma scheduled for total pancreatectomy.

Clinical Question**:** Please provide follow-up imaging of the findings.

Radiology Request Form 5 (Abdominal Oncology)

German (Original Radiology Request Form):

Anamnese: V.a. Pankreaskarzinom DD Autoimmunpankreatitis

Fragestellung: Lokalbefund? Lebermetastasen?

English:

Medical History**:** Suspected pancreatic carcinoma vs. autoimmune pancreatitis.

Clinical Question**:** Local findings? Liver metastases?

Radiology Request Form 6

German (Original Radiology Request Form):

Anamnese: Verdacht auf Primär sklerosierende Cholangitis. Patient mit Colitis Ulcerosa.

Fragestellung: PSC?

English:

Medical History**:** Suspected primary sclerosing cholangitis. Patient with ulcerative colitis.

Clinical Question**:** PSC?

Radiology Request Form 7

German (Original Radiology Request Form):

Anamnese: Alkalische Phosphatase und Gamma-GT erhöht.

Fragestellung: PSC?

English:

Medical History**:** Elevated alkaline phosphatase and gamma-GT.

Clinical Question**:** PSC?

Radiology Request Form 8

German (Original Radiology Request Form):

Anamnese: Dilatierte Gallengänge

Fragestellung: Erbete Korrelation bei sonographisch v.a. linksseitig zentral

dilatierte Gallengänge.

English:

Medical History**:** Dilated bile ducts

Clinical Question**:** Correlation requested with sonographic suspicion of centrally dilated bile ducts, predominantly on the left side.

Radiology Request Form 9

German (Original Radiology Request Form):

Anamnese: Anal-Ca. ED 12/22 seitdem nicht behandelt, HIV Stadium B/C3

Fragestellung: Staging.

English:

Medical History**:** Anal cancer, diagnosed 12/22, not treated since. HIV stage B/C3.

Clinical Question**:** Staging.

Radiology Request Form 10

German (Original Radiology Request Form):

Anamnese: Junge Patientin mit fibroiden, angiomatösem Histiozytom gluteal rechts. Strukturierte Nachsorge. Restaging nach primärer Resektion am 16.02.23.

Fragestellung: Staging. Filialisierung? Lokalrezidiv?

English:

Medical History**:** Young female patient with fibroid, angiomatous histiocytoma in the right gluteal region. Structured follow-up. Restaging after primary resection on 16.02.23.

Clinical Question**:** Staging. Metastasis? Local recurrence?

Radiology Request Form 11

German (Original Radiology Request Form):

Anamnese: Junge Patientin mit fibroiden, angiomatösem Histiozytom gluteal rechts. Strukturierte Nachsorge. Restaging nach primärer Resektion am 16.02.23.

Fragestellung: Staging. Filialisierung? Lokalrezidiv?

English:

Medical History**:** Young female patient with fibroid, angiomatous histiocytoma in the right gluteal region. Structured follow-up. Restaging after primary resection on 16.02.23.

Clinical Question**:** Staging. Metastasis? Local recurrence?

Radiology Request Form 12

German (Original Radiology Request Form):

Anamnese: Nicht abheilendes Rektumulcus.

Fragestellung: Verlaufskontrolle.

English:

Medical History**:** Non-healing rectal ulcer.

Clinical Question**:** Follow-up evaluation.

Radiology Request Form 13

German (Original Radiology Request Form):

Anamnese: Verdacht auf Rektumkarzinom.

Fragestellung: Staging.

English:

Medical History**:** Suspected rectal carcinoma.

Clinical Question**:** Staging

Radiology Request Form 14

German (Original Radiology Request Form):

Anamnese: V.a. abdominale Echinokokkose. Zustand nach Echinokokken-Zysten OP 2015 mit Entfernung einer hepatischen Zystenformation.

Fragestellung: MR-morphologische Korrelation.

English:

Medical History**:** Suspected abdominal echinococcosis. Status post echinococcal cyst surgery in 2015 with removal of a hepatic cystic lesion.

Clinical Question**:** MR-morphological correlation.

Radiology Request Form 15

German (Original Radiology Request Form):

Anamnese: Seit Wochen Schmerzen im linken Unterbauch. Verdachtr auf Tendinitis der Iliopsoas-Sehne links DD Divertikulose.

Fragestellung: Fokus im linken Unterbauch bzw. Malignität im gesamten

Abdomen und Leber?

English:

Medical History**:** Left lower abdominal pain for several weeks. Suspected iliopsoas tendonitis on the left, differential diagnosis: diverticulosis.

Clinical Question**:** Inflammatory focus in the left lower abdomen or malignancy in the entire abdomen and liver?

Radiology Request Form 16

German (Original Radiology Request Form):

Anamnese: Metastasiertes Mammakarzinom (ossär, pulmonal und hepatisch). Palbociclib bei schweren NW (Depression) seit 08/2019 abgesetzt.

Fragestellung: Restaging

English:

Medical History**:** Metastatic breast cancer (osseous, pulmonary, and hepatic). Palbociclib discontinued since 08/2019 due to severe side effects (depression).

Clinical Question**:** Restaging.

Radiology Request Form 17

German (Original Radiology Request Form):

Anamnese: Nachsorge bei retroperitonealem Sarkom rechts mit Zustand nach R0 Resektion 06.10.2022. Systematische retroperitoneale Kompartmentresektion rechts (Adrenalektomie, Nephrektomie, Hemikolektomie rechts) mit Diskontinuitätsresektion (endständiges Ileostoma).

Fragestellung: Nachsorge.

English:

Medical History: Follow-up after right-sided retroperitoneal sarcoma with R0 resection on 06.10.2022. Systematic right retroperitoneal compartment resection (adrenalectomy, nephrectomy, right hemicolectomy) with discontinuity resection (end ileostomy).

Clinical Question: Follow-up.

Radiology Request Form 18

German (Original Radiology Request Form):

Anamnese: Gesicherte Leberzirrhose.

Fragestellung: Hepatozelluläres Karzinom?

English:

Medical History: Confirmed liver cirrhosis.

Clinical Question: Hepatocellular carcinoma?

Radiology Request Form 19

German (Original Radiology Request Form):

Anamnese: HCC Segment V bei chronischer Hepatitis B.

Fragestellung: Aktuelles MRT der Leber vor geplanter OP.

English:

Medical History: HCC in segment V in the setting of chronic hepatitis B.

Clinical Question: Current liver MRI prior to planned surgery.

Radiology Request Form 20

German (Original Radiology Request Form):

Anamnese: Bild der Fettleberzirrhose.

Suspekte echoarme Läsion (neu) in S V mit 1,3 cm Größe, im CEUS hochgradig HCC-suspekt (CEUS-LI-RADS 5)

Leberzirrhose Child A auf dem Boden einer chronischen Hepatitis B.

Fragestellung: Staging bei V.a. HCC

English:

Medical History**:** Imaging consistent with fatty liver cirrhosis.
New hypoechoic lesion in segment V measuring 1.3 cm, highly suspicious for HCC on CEUS (CEUS LI-RADS 5).
Liver cirrhosis Child-Pugh A due to chronic hepatitis B.

Clinical Question**:** Staging for suspected HCC.

Radiology Request Form 21

German (Original Radiology Request Form):

Anamnese: Extern nachgewiesenes HCC histologisch, Bildgebung allerdings insuffizient zur Planung eventueller Lokaltherapie.

Fragestellung: Staging.

English:

Medical History**:** Histologically confirmed HCC diagnosed externally; imaging insufficient for planning potential local therapy.

Clinical Question**:** Staging.

Radiology Request Form 22

German (Original Radiology Request Form):

Anamnese: Sonographisch Zeichen der Fettleberzirrhose.

Neu aufgetretenes echoarmes Areal perivesikal DD Anderverfettungsareal DD maligne Raumforderung.

Ubiquitär morphologisch gleichartige echoarme Leberläsionen bis max. 7 mm Größe (idem zu den Vorbefunden).

Fragestellung: HCC?

English:

Medical History**:** Sonographic signs of fatty liver cirrhosis.
New hypoechoic area near the gallbladder—differential diagnosis: focal fat sparing vs. malignant lesion.

Multiple morphologically similar hypoechoic liver lesions throughout, up to 7 mm in size (unchanged from previous findings).

Clinical Question**:** HCC?

Radiology Request Form 23

German (Original Radiology Request Form):

Anamnese: Leberzirrhose

Fragestellung: Evaluation für LTX Listung

English:

Medical History**:** Liver cirrhosis

Clinical Question**:** Evaluation for liver transplantation (LTX) listing.

Radiology Request Form 24

German (Original Radiology Request Form):

Anamnese: Autoimmunhepatitis, HCC-Surveillance

Fragestellung: HCC?

English:

Medical History**:** Autoimmune hepatitis, under HCC surveillance

Clinical Question**:** HCC?

Radiology Request Form 25

German (Original Radiology Request Form):

Anamnese: Sonographisch Zeichen der Leberzirrhose mit multiplen flau abgrenzbaren echoreichen und echoarmen Herden (<1 cm). Zudem eine relativ scharf abgrenzbare echoreiche Läsion im rechten Leberlappen; B-bildmorphologisch suspekte Läsionen nicht sicher auszuschließen.

Fragestellung: HCC?

English:

Medical History**:** Sonographic signs of liver cirrhosis with multiple poorly defined hyperechoic and hypoechoic lesions (<1 cm). Additionally, a relatively well-defined hyperechoic lesion in the right liver lobe; suspicious lesions cannot be definitively excluded based on B-mode imaging.

Clinical Question**:** HCC?

4. Muskuloskeletal imaging

Radiology Request Form 1

German (Original Radiology Request Form):

Anamnese: Zervikobrachialgie rechts mit intermittierender Kraftlosigkeit rechts.

Fragestellung: Ausschluss NPP.

English:

Medical History**:** Right-sided cervicobrachialgia with intermittent weakness on the right side.

Clinical Question**:** Rule out nerve root compression (NRC).

Radiology Request Form 2

German (Original Radiology Request Form):

Anamnese: Bekannte MS. Nun Taubheitsgefühl beider Hände. Bekannter Herd C5/6.

Fragestellung: Ausschluss Karpaltunnelsyndrom

English:

Medical History**:** Known multiple sclerosis. Now presenting with numbness in both hands. Known lesion at C5/6.

Clinical Question**:** Rule out carpal tunnel syndrome.

Radiology Request Form 3

German (Original Radiology Request Form):

Anamnese: Abszedierung des linken M. iliopsoas 01/24

- Klinik: linksseitiger Unterbauchschmerz, Kraftminderung Hüftflexion, reduzierte Beweglichkeit linke Hüfte

- Labor: CRP 316 mg/l

- BKpp versandt

- CT-Abdomen (extern): Befund vereinbar mit Abszedierung im linksseitigen M. psoas bis in den M. iliacus. Periaortale Flüssigkeitsansammlung m. teils unscharfer Abbildung der Aortenwand infrarenal

- UCH: keine akute OP-Indikation, soll in der Frühbesprechung am 31.01. besprochen werden

- Therapie: Piperacillin/Tazobactam seit 31.01.

- Prozedere: CT-gesteuerte Drainage 01.02.

Fragestellung: Ausgeprägter Psoasabszess linksseitig, Bewegungseinschränkung,

massiv erhöhte Inflammationsparameter. Entsprechend Empfehlung Ortho dringend LWS Becken mit Frage nach Spondylodiszitis

English:

Medical History:

- Abscess formation in the left iliopsoas muscle (01/24)
- Clinical presentation: left lower abdominal pain, reduced strength in hip flexion, limited mobility of the left hip
- Laboratory findings: CRP 316 mg/L
- Blood cultures sent
- External abdominal CT: findings consistent with abscess formation in the left psoas muscle extending into the iliacus muscle; periaortic fluid collection with partially blurred imaging of the infrarenal aortic wall
- Surgical consult: no indication for emergency surgery; case to be discussed in the early meeting on 31.01
- Therapy: Piperacillin/Tazobactam since 31.01
- Planned procedure: CT-guided drainage on 01.02

Clinical Question: Extensive left-sided psoas abscess, restricted mobility, markedly elevated inflammatory markers. As per orthopedic recommendation, urgent evaluation of lumbar spine and pelvis for suspected spondylodiscitis.

Radiology Request Form 4

German (Original Radiology Request Form):

Anamnese: L1 Syndrom rechts. V.a. Bandscheibenprolaps.

Fragestellung: NPP/Stenosen.

English:

Medical History**:** L1 syndrome on the right. Suspected disc herniation.

Clinical Question**:** Nerve root compression (NRC) / spinal stenosis?

Radiology Request Form 5

German (Original Radiology Request Form):

Anamnese: Fraktur BWK 3 mit Sternumfraktur.

Fragestellung: Ausschluss B-Verletzung.

English:

Medical History**:** Fracture of thoracic vertebra T3 with sternal fracture.

Clinical Question**:** Rule out posterior (B-type) spinal injury.

Radiology Request Form 6

German (Original Radiology Request Form):

Anamnese: Neuroendokriner Tumor mit unbekanntem Primarius (NET G2, Ki67-Index 10-15%), DD Pankreas DD typisches Karzinoid, ED 05/2022 mit osteolytischer Metastase im linken proximalen Femur.

Fragestellung: Kurzfristig konstante, gegenüber 06/2022 größenprogrediente osteolytische Läsion rechtsseitig im Wirbelbogen von BWK 4 mit intakter angrenzender Corticalis und ohne Nuklidmehrbelegung in der PET/CT von 03/2023, unklar, Metastase aufgrund der Dynamik nicht auszuschließen. Ergänzende MRT der oberen BWS empfohlen.

English:

Medical History**:** Neuroendocrine tumor (NET G2, Ki-67 index 10–15%) with unknown primary, differential diagnosis: pancreas or typical carcinoid. Diagnosed 05/2022 with an osteolytic metastasis in the left proximal femur.

Clinical Question**:** Recently stable but progressively enlarging osteolytic lesion in the right vertebral arch of T4 compared to 06/2022, with intact adjacent cortex and no increased tracer uptake in PET/CT from

03/2023. Metastasis cannot be excluded due to dynamic changes. Additional MRI of the upper thoracic spine recommended.

Radiology Request Form 7

German (Original Radiology Request Form):

Anamnese: Taubheit und Parästhesien in den Händen bds. seit 2 Wochen zunehmend. Außerdem Nackenschmerzen. Leichtgradige Paresen der Fingerbeugung und Streckung.

Fragestellung: HWS Prozess? C7 Affektion? Myelitis?

English:

Medical History**:** Numbness and paresthesia in both hands, progressively worsening over the past 2 weeks. Also experiencing neck pain. Mild weakness in finger flexion and extension.

Clinical Question**:** Cervical spine pathology? C7 involvement? Myelitis?

Radiology Request Form 8

German (Original Radiology Request Form):

Anamnese: Rasch progrediente afferent-sensible Gangstörung DD Hinterstrangaffektion. Begonnen habe dies mit Missempfindungen im linken Fuß sowie das rechte Bein aktuell zunehmend, sowie auch beidseitige, unsymmetrische, brennende Missempfindungen vor der klinisch-neurologischen Untersuchung zeigte sich eine ausgeprägte, spastisch-ataktische Gangstörung bei zudem polyneuropathischen Syndrom. Elektrophysiologisch zeigte sich eine Affektion der sensiblen Bahnen von beiden Armen und hochgradig von beiden Beinen. Monoklonale Gammopathie vom Typ IgG-kappa. Arterielle Hypertonie. Nikotinabusus (80 pack years).

Fragestellung: MR-morphologisches Korrelat? Hinweis auf Spinalkanalstenose?

mögliche Raumforderung?

English:

Medical History**:** Rapidly progressive sensory ataxic gait disturbance, differential diagnosis: posterior column involvement. Initially presented with paresthesia in the left foot, currently progressing in the right leg as well, along with bilateral, asymmetrical burning sensations. Prior to clinical-neurological evaluation, there was a marked spastic-ataxic gait disturbance in the context of a polyneuropathic syndrome. Electrophysiology showed involvement of the sensory pathways in both arms and severe

involvement in both legs. Monoclonal gammopathy of IgG-kappa type. Arterial hypertension. History of nicotine abuse (80 pack years).

Clinical Question**:** MR-morphological correlate? Evidence of spinal canal stenosis? Possible Malignancy?

Radiology Request Form 9

German (Original Radiology Request Form):

Anamnese: V. a. eingeklemmten Meniskusriss

Fragestellung: Meniskusläsion?

English:

Medical History**:** Suspected incarcerated meniscal tear

Clinical Question**:** Meniscal lesion?

Radiology Request Form 10

German (Original Radiology Request Form):

Anamnese: Schmerzen nach Belastung beim Fußball im inneren oberen Bereich des linken Knies. Kein Trauma erinnerlich.

Fragestellung: Zum Ausschluss Innenmeniskusläsion links.

English:

Medical History**:** Pain after exertion while playing football, located in the inner upper area of the left knee. No recalled trauma.

Clinical Question**:** To rule out medial meniscus lesion on the left.

Radiology Request Form 11

German (Original Radiology Request Form):

Anamnese: Z.n. Fahrradsturz. Schmerzen im Knie rechts, innen superior betont.

Fragestellung: Traumafolge.

English:

Medical History**:** Status post bicycle accident. Pain in the right knee, predominantly superior medial.

Clinical Question**:** Consequences of trauma?

Radiology Request Form 12

German (Original Radiology Request Form):

Anamnese: Unklare Raumforderung distaler Oberschenkel rechts lateral.

Fragestellung: Präoperative Abklärung bei V.a. Liposarkom.

English:

Medical History**:** Unclear mass in the distal right lateral thigh.

Clinical Question**:** Preoperative evaluation for suspected liposarcoma.

Radiology Request Form 13

German (Original Radiology Request Form):

Anamnese: Knieschmerzen rechts, IM Zeichen positiv

Fragestellung: IM Riss?

English:

Medical History**:** Right knee pain, positive McMurray sign.

Clinical Question**:** Inner meniscus tear?

Radiology Request Form 14

German (Original Radiology Request Form):

Anamnese: Autoinflammatorisches Syndrom, bislang ohne eindeutige diagnostische Zuordnung. Klinik: intermittierendes Fieber, Erytheme: schmetterlingsförmig im Gesicht, Hals, Handrücken, Knie, Arthralgien, dauerhaft erhöhte Inflammationsparameter (CRP 10-40 mg/l).

Fragestellung: Osteoymelitits Knie links? Knieempyem? Serositis?

English:

Medical History**:** Autoinflammatory syndrome without clear diagnostic classification to date. Clinical presentation includes intermittent fever, erythema (butterfly-shaped on the face, as well as on the neck, backs of the hands, and knees), arthralgia, and persistently elevated inflammatory markers (CRP 10–40 mg/L).

Clinical Question**:** Osteomyelitis of the left knee? Knee empyema? Serositis?

Radiology Request Form 15

German (Original Radiology Request Form):

Anamnese: Knieschmerzen, Raumforderung dorsomedialer distaler Femur links.

Fragestellung: Weiterführende Abklärung der knöchernen Läsion im distalen Femur.

English:

Medical History**:** Knee pain with a mass in the dorsomedial aspect of the distal left femur.

Clinical Question**:** Further evaluation of the bony lesion in the distal femur.

Radiology Request Form 16

German (Original Radiology Request Form):

Anamnese: Akute lymphoblastische Leukämie, a.e. common-B-ALL. Knocheninfarkte Hüfte bds li > re

Fragestellung: Knieschmerzen links. Pathologie?

Knocheninfarkte im Rahmen der Behandlung der Grunderkrankung?

English:

Medical History**:** Acute lymphoblastic leukemia, most likely common B-ALL. Bone infarctions in both hips, more pronounced on the left.

Clinical Question**:** Left knee pain. Pathology?
Bone infarctions as a result of treatment for the underlying disease?

Radiology Request Form 17

German (Original Radiology Request Form):

Anamnese: z.A. neoplastischer Prozess Z.n. dislozierter Claviculafraktur rechts

Fragestellung: Malignität?

English:

Medical History**:** Suspected neoplastic process. Status post displaced right clavicle fracture.

Clinical Question**:** Malignancy?

Radiology Request Form 18

German (Original Radiology Request Form):

Anamnese: Solitäres Plasmozytom im linken Glenoid, ED 12/22, Z.n. Radiatio bis 03/23

Fragestellung: Nachsorge mit Frage nach Therapieansprechen.

English:

Medical History**:** Solitary plasmacytoma in the left glenoid, diagnosed 12/22, status post radiation therapy until 03/23.

Clinical Question**:** Follow-up to assess treatment response.

Radiology Request Form 19

German (Original Radiology Request Form):

Anamnese: Dermatofibrosarkoma protuberans, Stadium T1cN0cM0 GX ED 01/2018. Erfolgte Resektion.

Fragestellung: Verlaufskontrolle.

English:

Medical History**:** Dermatofibrosarcoma protuberans, stage T1cN0cM0 GX, diagnosed 01/2018. Resection performed.

Clinical Question**:** Follow-up evaluation.

Radiology Request Form 20

German (Original Radiology Request Form):

Anamnese: Unklare Raumforderung Clavicula rechts. Zusammenfassend sei die Patientin initial mit Beschwerden im Bereich des rechten Schlüsselbeins bei und nach Belastung im Sommer 2022 aufgefallen. Hier sei bereits eine radiologische Bildgebung beim Orthopäden erfolgt, welcher keine Fraktur festgestellt habe. Im Verlauf seien die Beschwerden dann regredient gewesen. Im Dezember 2022 dann eine erneute Zunahme der Beschwerden, sodass am 13.3.2023 ein MRT erfolgt sei, bei dem man eine ödematöse Reaktion der Clavicula mit V. a. Fraktur festgestellt habe.

Fragestellung: Dignität?

English:

Medical History**:** Unclear mass in the right clavicle. The patient initially presented with load-dependent discomfort in the right clavicle area in the summer of 2022. Radiological imaging by an orthopedic specialist at that time showed no fracture. Symptoms subsided over time. In December 2022, symptoms worsened again, leading to MRI on 13.03.2023, which revealed an edematous reaction in the clavicle with suspected fracture.

Clinical Question**:** Nature of the lesion (benign vs. malignant)?

Radiology Request Form 21

German (Original Radiology Request Form):

Anamnese: Seit Impfung schmerzhafte Bewegungseinschränkung in linkem Arm. Druckschmerz auf Acromicolaviculargelenk. Impingment-Syndrom der Schulter.

Fragestellung: Erbitte MRT Schulter links, bitte Beurteilung der Impfstelle, diese sollte markiert werden.

English:

Medical History**:** Painful restriction of movement in the left arm since vaccination. Tenderness over the acromioclavicular joint. Shoulder impingement syndrome.

Clinical Question**:** Requesting MRI of the left shoulder, including assessment of the injection site, which should be marked.

Radiology Request Form 22

German (Original Radiology Request Form):

Anamnese: Unklares Krankheitsbild mit Zytopenie seit 9 Monaten und nun ausgeprägte Muskelschmerzen, Sturzsenkung, Fieber

Fragestellung: Hinweis auf Osteitis oder Fasciitis?

English:

Medical History**:** Unclear clinical picture with cytopenia for 9 months, now presenting with severe muscle pain, elevated ESR, and fever.

Clinical Question**:** Evidence of osteitis or fasciitis?

Radiology Request Form 23

German (Original Radiology Request Form):

Anamnese: Raumforderung Schulterblatt Oberrand Scapula

Fragestellung: Darstellung Tumor.

English:

Medical History**:** Mass at the superior border of the scapula.

Clinical Question**:** Tumor visualization.

Radiology Request Form 24

German (Original Radiology Request Form):

Anamnese: Lipom seit ungefähr einem Jahr bestehend, seit gestern schmerzend, weich, verschieblich ca 5 cm Durchmesser.

Fragestellung: Entzündung? Tumoröse Entartung?

English:

Medical History**:** Lipoma present for approximately one year, now painful since yesterday. Soft, mobile, approximately 5 cm in diameter.

Clinical Question**:** Inflammation? Tumorous transformation?

Radiology Request Form 25

German (Original Radiology Request Form):

Anamnese: Lumbosdemoralgie rechts und Koxalgie rechts

Fragestellung: NPP?

English:

Medical History**:** Right-sided lumbosacral pain and right hip pain**.**

Clinical Question**:** Nerve root compression (NRC)?

Supplementary material 3: Number of missing sequences listed by case and reader.

Case	Area	GPT-4	Reader 1	Reader 2
1	Cardiac	0	0	0
2	Cardiac	0	0	0
3	Cardiac	0	0	0
4	Cardiac	0	0	0
5	Cardiac	0	0	0
6	Cardiac	0	0	1
7	Cardiac	0	0	0
8	Cardiac	0	0	0
9	Cardiac	0	0	0
10	Cardiac	0	0	0
11	Cardiac	0	2	0
12	Cardiac	1	0	1
13	Cardiac	0	0	0
14	Cardiac	0	0	0
15	Cardiac	1	0	0
16	Cardiac	0	0	0
17	Cardiac	0	1	0
18	Cardiac	0	1	0
19	Cardiac	1	1	0
20	Cardiac	1	1	0
21	Cardiac	1	1	0
22	Cardiac	1	2	1
23	Cardiac	0	0	0
24	Cardiac	0	0	0
25	Cardiac	0	0	0
26	Neuro	0	0	0
27	Neuro	0	0	0
28	Neuro	0	0	0
29	Neuro	0	0	0
30	Neuro	0	0	3
31	Neuro	3	3	0
32	Neuro	0	0	0
33	Neuro	0	0	0
34	Neuro	0	0	0
35	Neuro	0	0	0
36	Neuro	0	0	0
37	Neuro	0	1	0
38	Neuro	2	1	3
39	Neuro	0	3	0
40	Neuro	4	0	4
41	Neuro	0	4	0
42	Neuro	0	3	0
43	Neuro	0	0	0
44	Neuro	0	0	0

45	Neuro	0	3	0
46	Neuro	0	0	0
47	Neuro	0	0	0
48	Neuro	0	0	0
49	Neuro	0	0	1
50	Neuro	0	0	1
51	Oncology	1	2	0
52	Oncology	1	2	0
53	Oncology	1	2	0
54	Oncology	1	2	0
55	Oncology	1	2	0
56	Oncology	0	1	0
57	Oncology	0	1	0
58	Oncology	0	1	1
59	Oncology	0	0	0
60	Oncology	0	0	0
61	Oncology	1	1	0
62	Oncology	1	1	1
63	Oncology	1	1	1
64	Oncology	1	1	1
65	Oncology	2	2	4
66	Oncology	5	6	2
67	Oncology	1	2	1
68	Oncology	1	1	1
69	Oncology	1	1	1
70	Oncology	1	1	1
71	Oncology	1	1	1
72	Oncology	1	1	1
73	Oncology	1	1	1
74	Oncology	1	1	1
75	Oncology	1	1	1
76	MSK	1	0	0
77	MSK	3	3	3
78	MSK	2	1	1
79	MSK	1	0	0
80	MSK	1	0	0
81	MSK	3	1	1
82	MSK	2	1	0
83	MSK	2	2	1
84	MSK	3	0	0
85	MSK	4	1	1
86	MSK	2	2	2
87	MSK	2	0	0
88	MSK	3	0	0
89	MSK	3	2	1
90	MSK	2	1	1
91	MSK	2	2	2

92	MSK	1	1	0
93	MSK	3	2	2
94	MSK	2	3	1
95	MSK	1	1	1
96	MSK	2	2	2
97	MSK	2	1	0
98	MSK	1	1	0
99	MSK	2	2	1
100	MSK	3	0	0

Supplementary material 4: Number of unnecessary sequences listed by case and reader.

Case	Area	GPT-4	Reader 1	Reader 2
1	Cardiac	2	1	0
2	Cardiac	1	1	0
3	Cardiac	1	1	0
4	Cardiac	1	1	0
5	Cardiac	0	1	0
6	Cardiac	0	1	0
7	Cardiac	0	0	0
8	Cardiac	1	1	0
9	Cardiac	0	1	0
10	Cardiac	0	0	0
11	Cardiac	0	2	0
12	Cardiac	0	1	0
13	Cardiac	0	1	0
14	Cardiac	0	1	0
15	Cardiac	0	1	0
16	Cardiac	0	1	0
17	Cardiac	0	1	0
18	Cardiac	0	1	0
19	Cardiac	0	1	0
20	Cardiac	0	1	0
21	Cardiac	0	1	1
22	Cardiac	0	1	0
23	Cardiac	0	1	0
24	Cardiac	0	1	0
25	Cardiac	0	1	0
26	Neuro	1	0	0
27	Neuro	1	0	0
28	Neuro	1	0	1
29	Neuro	2	0	0
30	Neuro	1	0	0
31	Neuro	1	3	1
32	Neuro	0	0	0
33	Neuro	1	0	0
34	Neuro	1	0	0
35	Neuro	1	1	0
36	Neuro	2	0	0
37	Neuro	1	4	1
38	Neuro	1	3	3
39	Neuro	0	2	0
40	Neuro	5	0	4
41	Neuro	0	4	1
42	Neuro	1	3	0
43	Neuro	1	0	0

44	Neuro	1	0	1
45	Neuro	2	3	0
46	Neuro	0	0	0
47	Neuro	1	0	0
48	Neuro	1	1	0
49	Neuro	0	0	2
50	Neuro	1	0	1
51	Oncology	0	0	0
52	Oncology	0	1	0
53	Oncology	0	1	0
54	Oncology	0	0	0
55	Oncology	0	0	0
56	Oncology	0	0	1
57	Oncology	0	0	1
58	Oncology	0	0	1
59	Oncology	0	0	0
60	Oncology	0	0	0
61	Oncology	1	1	1
62	Oncology	1	1	0
63	Oncology	1	1	1
64	Oncology	1	1	1
65	Oncology	0	0	3
66	Oncology	5	3	1
67	Oncology	0	0	0
68	Oncology	0	0	0
69	Oncology	0	0	0
70	Oncology	0	0	0
71	Oncology	0	0	0
72	Oncology	0	0	0
73	Oncology	0	0	0
74	Oncology	0	0	0
75	Oncology	0	0	0
76	MSK	1	0	0
77	MSK	2	2	3
78	MSK	2	1	0
79	MSK	1	0	0
80	MSK	0	0	0
81	MSK	2	1	0
82	MSK	1	1	0
83	MSK	2	2	1
84	MSK	3	0	1
85	MSK	1	0	0
86	MSK	2	1	1
87	MSK	2	0	0
88	MSK	3	1	1
89	MSK	2	1	0
90	MSK	1	2	0

91	MSK	1	0	0
92	MSK	2	2	1
93	MSK	0	0	0
94	MSK	1	2	1
95	MSK	2	1	1
96	MSK	2	2	2
97	MSK	0	0	0
98	MSK	1	1	0
99	MSK	2	1	1
100	MSK	1	0	0
